# Unraveling the Influence of Litter Size, Maternal Care, Exercise, and Aging on Neurobehavioral Plasticity and Dentate Gyrus Microglia Dynamics in Male Rats

**DOI:** 10.3390/brainsci14050497

**Published:** 2024-05-15

**Authors:** Lane Viana Krejcová, João Bento-Torres, Daniel Guerreiro Diniz, Antonio Pereira, Manuella Batista-de-Oliveira, Andreia Albuquerque Cunha Lopes de Morais, Rosângela Figueiredo Mendes-da-Silva, Ricardo Abadie-Guedes, Ângela Amâncio dos Santos, Denise Sandrelly Lima, Rubem Carlos Araujo Guedes, Cristovam Wanderley Picanço-Diniz

**Affiliations:** 1 Neurodegeneration and Infection Research Laboratory, João de Barros Barreto Universitary Hospital, Institute of Biological Sciences, Federal University of Pará, Belém 66050-160, Pará, Brazil; 2 Postgraduate Program in Oncology and Medical Sciences, João de Barros Barreto Universitary Hospital, Federal University of Pará, Belém 66075-110, Pará, Brazil; 3 Electron Microscopy Laboratory, Evandro Chagas Institute, Belém 66093-020, Pará, Brazil; 4 Naíde Teodósio Nutrition Physiology Laboratory, Department of Nutrition, Federal University of Pernambuco, Recife 50670-901, Pernambuco, Brazil

**Keywords:** exercise, litter size, maternal care, age-related memory impairments, object recognition, dentate gyrus, microglia

## Abstract

This study explores the multifaceted influence of litter size, maternal care, exercise, and aging on rats’ neurobehavioral plasticity and dentate gyrus microglia dynamics. Body weight evolution revealed a progressive increase until maturity, followed by a decline during aging, with larger litters exhibiting lower weights initially. Notably, exercised rats from smaller litters displayed higher body weights during the mature and aged stages. The dentate gyrus volumes showed no significant differences among groups, except for aged sedentary rats from smaller litters, which exhibited a reduction. Maternal care varied significantly based on litter size, with large litter dams showing lower frequencies of caregiving behaviors. Behavioral assays highlighted the detrimental impact of a sedentary lifestyle and reduced maternal care/large litters on spatial memory, mitigated by exercise in aged rats from smaller litters. The microglial dynamics in the layers of dentate gyrus revealed age-related changes modulated by litter size and exercise. Exercise interventions mitigated microgliosis associated with aging, particularly in aged rats. These findings underscore the complex interplay between early-life experiences, exercise, microglial dynamics, and neurobehavioral outcomes during aging.

## 1. Introduction

Maternal care is a pivotal factor in shaping offspring development, encompassing a myriad of behavioral, physiological, and neurobiological processes [1]. A series of experiments dedicated to measuring the impact of reduced maternal care in rats has demonstrated important detrimental effects on central nervous system (CNS) development and adult brain function [2,3]. Such impacts on brain development were also observed across nonhuman primates and humans [4,5,6,7]. Among these processes, the intricate interplay between maternal care, brain inflammation, and microglial dynamics has emerged as a focal point in understanding neurodevelopmental outcomes [8,9].

Microglia, the resident immune cells of the central nervous system, play multifaceted roles in modulating brain homeostasis, as well as synaptic pruning, and have profound influences on microglial function and neuroinflammatory states, thereby impacting offspring’s neurobiological trajectories [10,11,12]. Microglia exhibit remarkable plasticity and responsiveness to environmental stimuli, including maternal care quality and quantity. Moreover, microglial activation and subsequent inflammatory responses are intricately linked to neurodevelopmental outcomes, with implications for synaptic pruning, neurogenesis, and cognitive function [8,9].

Maternal care encompasses a spectrum of nurturing behaviors, including grooming, nursing, and protection, crucial for offspring survival and development [1]. Studies have elucidated the crucial role of maternal care in regulating microglial morphology, distribution, and activity during critical periods of neurodevelopment. Moreover, maternal care has been implicated in modulating neuroinflammatory processes, with implications for neuroplasticity and cognitive function in the offspring [13,14].

Intriguingly, maternal care dynamics are not uniform across reproductive contexts, with litter size representing a critical determinant. Variations in litter size introduce differential demands on maternal resources and caregiving behaviors, thereby influencing offspring outcomes. While larger litter sizes often necessitate increased maternal investment, smaller litter sizes may afford heightened maternal attention and resources per offspring. Consequently, investigating the neurobiological consequences of varying litter sizes provides a unique lens into the interplay between maternal care, microglial function, and neuroinflammation.

In rodent models, studies have revealed lasting metabolic changes and epigenetic implications associated with extreme litter sizes, ranging from as few as 3 or 4 pups per litter [15,16,17] to as high as 16 to 18 pups per litter [18,19,20]. A profound influence of litter size on the quantity of maternal care allocated per pup has been demonstrated, exhibiting notable effects on metabolic traits, behavioral patterns, and immune responses [21,22].

Nonetheless, the understanding of the long-term effects of litter size on the interface between the neural and immune systems remains limited [21,23], particularly on the CNS immune cells of adult and aged animals [24]. While existing evidence highlights the impact of litter size on corticosterone levels during early postnatal stages [18,25], substantial gaps persist in our knowledge regarding the enduring consequences of microglial response in the CNS [26,27,28,29].

Furthermore, recent studies have begun elucidating the role of voluntary exercise as a modulator of microglial activity and neuroinflammation in the context of maternal care. Exercise-induced alterations in microglial phenotype and function may interact with maternal care dynamics to shape offspring neurodevelopment [30,31]. This multifaceted enrichment approach fosters the expression of neurotrophins and prompts epigenetic modifications, thereby aiding in alleviating developmental deficits within the central nervous system (CNS) [32].

Voluntary exercise has been found to mitigate microglial proliferation and inhibit their activation within the hippocampus of stress-induced depressed rats [33]. Additionally, in aged animals, exercise promotes a pro-neurogenic phenotype in microglia [34], while moderate physical training has shown promise in mitigating the effects of perinatal undernutrition on the peripheral immune system [35]. Thus, unraveling the interconnected pathways linking maternal care, microglial activity, inflammation, and voluntary exercise holds promise for elucidating mechanisms underlying neurodevelopmental plasticity and resilience.

Our previous studies have underscored the dentate gyrus’ heightened susceptibility to the impacts of aging and sedentary lifestyles, particularly evident in rats raised in larger litters [29]. Furthermore, the hippocampus is implicated in both object recognition and spatial memory in both human and experimental models [36,37,38,39,40,41]. Neuroanatomical tracing has revealed intricate projections of perforant pathways onto dentate granule cells within the dentate gyrus, originating from layer two principal neurons of the entorhinal cortex (EC) and forming synapses at granule cell dendrites in the outer two-thirds of the molecular layer [42]. These projections are ipsilateral and arise exclusively from cortical layer two neurons [43]. Notably, the outer third of the molecular layer of the dentate gyrus corresponds to entorhinal projections involved in object recognition, while the middle third is associated with spatial learning and memory [39]. Given the relevance of these projections to object recognition and spatial memory, we have chosen to investigate microglial changes within the dentate gyrus layers.

Moreover, we have shown that environmental enrichment contributes to partially restoring age-related memory impairments and associated astroglia changes in mice [44,45]. This study examines the long-term effects of two different litter sizes (6 and 12 pups per dam) on the number and laminar distribution of microglia in the dentate gyrus. We also assess object recognition and spatial memory in adult mature rats (4 months old) and aged rats (23 months old) that either remained sedentary throughout their lives or underwent five weeks of exercise later in life.

Using the optical fractionator method, we quantified the number of microglia in the dentate gyrus to test the hypothesis that litter size and a sedentary lifestyle may influence both the quantity and distribution of microglia. We further investigated whether these microglial changes are associated with impairments in object recognition and spatial memory. Additionally, we explored whether five weeks of exercise later in life could counteract changes in microglial numbers and cognitive function.

## 2. Materials and Methods

All procedures undertaken in this investigation received approval from the institutional animal care committee of the Federal University of Pernambuco, Brazil. These procedures were conducted strictly with the “Principles of Laboratory Animal Care” outlined by the NIH (National Institutes of Health).

### 2.1. Experimental Groups

The study utilized offspring from an outbred colony of Wistar rats obtained from the Department of Nutrition at the Federal University of Pernambuco. Female Wistar rats were provided with standard rodent laboratory chow (Purina do Brazil Ltd., Vargeão, Brazil) and housed in groups of 2 or 3. Upon mating and gestation, the female Wistar rats typically gave birth to litters ranging from 7 to 12 pups. To study the effects of maternal care and suckling competition, we established two pup-to-dam ratios: 6:1, representing small litters (*n* = 20), and 12:1, representing large litters (*n* = 20). This adjustment was made within 48 h after birth.

Within the same 48 h window, pups from different litters were randomly redistributed among new litters. Offspring from various dams were pooled, mixed, and then randomly assigned to litters of six or twelve pups per dam. This method aimed to reduce the impact of genetic traits specific to individual litters, thereby focusing on the effects of litter size on maternal care and offspring development. All male pups were chosen and allocated among the litters. In contrast, female pups were included only if the number of male pups was insufficient to meet the predetermined litter size during lactation. Following weaning, exclusively male rats were chosen as experimental subjects.

**Figure 1:**
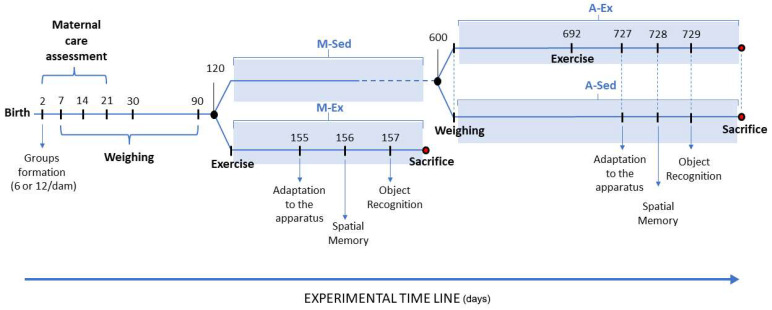
Timeline depicting the assessment of body weight and maternal care behaviors in pre- and post-weaned Wistar rats from small (6 pups/dam) and large litters (12/dam). M-Sed: mature adult rats leading a sedentary lifestyle; M-Ex: mature adult rats undergoing a regular exercise regimen; A-Sed: sedentary aged rats; A-Ex: aged rats undergoing a regular exercise regime.

The objective was to investigate whether litter size affects milk access competition and maternal care [46,47,48]. To achieve this, we meticulously observed and quantified the maternal care provided by each dam across small and large litters from the 3rd to the 21st postnatal day (weaning period). Additionally, body weights were recorded at various intervals to monitor the subjects’ growth under different experimental conditions. Figure 1 illustrates the timeline of the experimental procedures involving pre- and post-weaned Wistar rats.

### 2.2. Maternal Care Assessment

To assess the variation in maternal care between dams with large and small litters, we systematically observed maternal behavior using a predefined protocol. Each observation session lasted for one hour [49,50], with consistent monitoring at specific intervals. These observations occurred four times during the light phase (09:00, 11:00, 13:00, and 15:00) and twice during the dark phase of the light–dark cycle (19:00 and 21:00).

The behaviors recorded encompassed the following:

(a) Mother’s absence from pups.
(b) Mother engaging in licking/grooming behavior towards pups, including both general body grooming and specific attention to the anogenital region.
(c) Mother nursing pups in an arched-back or low-back posture, often referred to as the ‘blanket’ posture, where the mother lays over the pups.
(d) Mother assuming a passive posture, lying either on her back or side while the pups nurse.
(e) Nest-building behavior exhibited by the mother.
(f) Mother carrying or retrieving pups to the nest.
(g) Pups being away from the nest.
(h) Mother displaying passive behavior within the nest.
(i) Mother being away from pups while eating.

### 2.3. Environment, Exercise, and Sedentary Conditions

After the suckling period, all experimental cohorts were granted unrestricted access to an identical rodent standard laboratory chow diet (23% protein), Purina do Brazil Ltd. They were then housed in polypropylene cages (51 × 35.5 × 18.5 cm) in groups of 2 or 3 animals, ensuring an environment consistent with standard laboratory conditions: a 12 h light–dark cycle (lights on at 6 a.m.) and room temperature maintained at 23 ± 1 °C. These housing parameters remained consistent for all animals post-weaning until the time of sacrifice.

Our exercise protocol was chosen based on previous studies showing that 20 min daily running sessions, held three times per week over a four-week period of forced treadmill exercise at 60% of the animal’s maximal oxygen uptake, effectively improved spatial memory deficits in aged rats [51,52,53]. In this study, during either the 4-month or 17-month time frames, half of each experimental group (*n* = 10) began a progressive treadmill exercise routine for five weeks, strictly adhering to the detailed parameters outlined in Table 1. The treadmill utilized (Insight Equipamentos Ltd., Ribeirão Preto, São Paulo, Brazil) offered precise control over the duration and speed of the moving platform. As a control measure, the sedentary animals were transferred each morning to an inactive treadmill for an equivalent duration.

### 2.4. Behavioral Assessment and Testing Procedures

**Figure 2:**
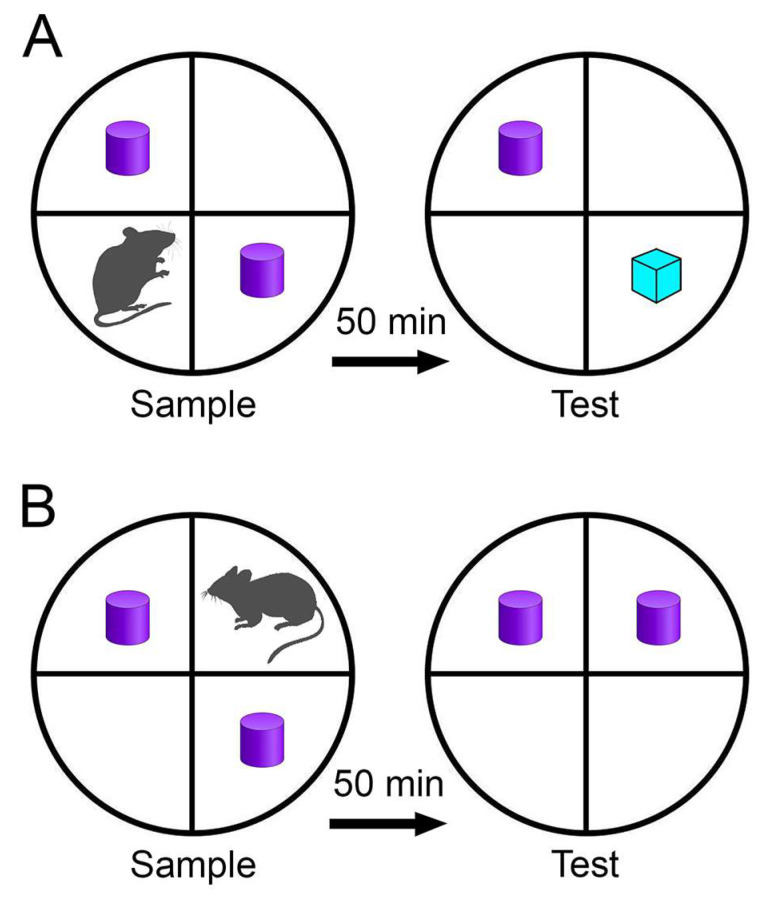
Schematic representation of the experimental setups for object recognition tests. (**A**). Object identity recognition: rats are anticipated to exhibit a preference for the “novel” object over the “familiar” one. Purple shapes represent the “familiar” object; blue shape represents the “novel” object. (**B**). Object placement recognition: rats are anticipated to display a preference for the “displaced” object over the “stationary” one. Adapted from [54].

Following the exercise regimen, all mature (4-month-old) and aged (23-month-old) rats, whether sedentary or engaged in exercise, underwent spatial memory and object recognition tests across all experimental groups. Figure 2 illustrates a schematic diagram of the object recognition and object placement apparatus, along with the corresponding test procedure. This study employed single-trial tests to evaluate object identity and placement recognition memories.

Apparatus Description and Test Protocol:

The apparatus utilized for the single-trial object recognition and spatial memory tests consisted of a painted, varnished wood circular container measuring 1 m in diameter. The floor featured marked lines to demarcate four quadrants, while the luminance at the center of the circular box floor was measured at 2.4 cd/m^2^. Detailed protocols and rationales for test selections have been previously documented [55,56,57].

The behavioral assessments spanned five days, with one day allocated for open-field habituation, two days for object habituation, and two days for testing purposes—dedicating one day to each specific test.

To minimize the influence of innate preferences for specific objects or materials, objects made from the same material yet featuring distinct geometries were chosen. These objects were deliberately selected for their ease of discrimination and offered similar potential for interaction [58]. Constructed from plastic, the objects varied in shapes, heights, and hues. Before introducing each rat into the arena, both the arena and objects underwent cleaning using 75% ethanol to minimize any discernible olfactory cues.

Testing Procedures:

Open-field habituation: Each animal explored the arena for 5 min without any objects present.

Object habituation: The animals encountered two identical objects (not used during test days) placed at distinct quadrants within the arena for 5 min; this was repeated three times with 50 min intervals.

One-trial recognition tests:

- Object identity test: The rats explored two identical objects during a 5 min sample trial. After a 50 min intermission, a second 5 min test trial introduced a “novel” object alongside a “familiar” one.
- Object identity recognition test: Mirroring the previous procedure, except one identical object was relocated during the test trial, termed the “displaced” object.

Behavioral Data Analysis:

Behavioral data underwent analysis using parametric statistics. A two-tailed t-test for dependent groups discerned significant differences in exploration time [59]. Performance was evaluated based on the exploration time for each object, expressed as a proportion of the total exploration time. Distinctions were deemed significant when one object’s exploration time exceeded 60% compared to the other. The threshold for significance was set at *p* < 0.05.

### 2.5. Immunohistochemical Analysis

After the behavioral tests, the rats were weighed and then anesthetized with an intraperitoneal injection of 2,2,2-tribromoethanol at a dose of 0.04 mL per gram of body weight. Once fully anesthetized, the rats underwent transcardial perfusion, starting with heparinized saline, followed by 4% paraformaldehyde in 0.1 M phosphate buffer (pH 7.2–7.4).

Following the perfusion, brain tissue sections were cut at a thickness of 70 µm using a Vibratome (Micron, Boise, ID, USA). These sections were then subjected to immunolabeling with a polyclonal antibody targeting ionized calcium-binding adapter molecule 1 (IBA-1), a marker for microglia, and macrophages. The antibody used was anti-Iba1 (catalog #019-19741; Wako Pure Chemical Industries Ltd., Osaka, Japan).

All the chemicals used in these procedures were sourced from Sigma-Aldrich (Poole, UK) or Vector Labs (Burlingame, CA, USA).

During immunolabeling, the free-floating sections underwent several treatments. Initially, they were pre-treated with 0.2 M boric acid (pH 9) at a temperature of 65–70 °C for 60 min to enhance antigen retrieval. Following this, the sections were washed in phosphate-buffered saline (PBS) and then immersed in 10% normal goat serum (Vector Laboratories, Newark, CA, USA) for 20 min to block non-specific binding. This step prepared the sections for IBA-1 immunolabeling.

Following this, the sections were incubated with anti-Iba1 (2 µg/mL in PBS) diluted in 0.1 M PBS (pH 7.2–7.4) for three days at 4 °C with continuous, gentle agitation. After primary incubation, the sections were washed and incubated overnight with a biotinylated secondary antibody (goat anti-rabbit for IBA-1, 1:250 in PBS, Vector Laboratories).

Subsequently, the sections were immersed in a 3% H_2_O_2_ solution in PBS to inhibit endogenous peroxidases. Following PBS washing, the sections were transferred to a solution of avidin–biotin–peroxidase complex (VECTASTAIN ABC kit; Vector Laboratories) for 1 h. After another round of washing, the sections were incubated in 0.1 M acetate buffer (pH 6.0) for 3 min and then developed in a solution comprising 0.6 mg/mL diaminobenzidine, 2.5 mg/mL ammonium nickel chloride, and 0.1 mg/mL glucose oxidase [60].

To validate the specificity of the immunohistochemical pattern, a negative control was conducted by omitting the primary antibody. This control revealed the absence of immunoreactivity across all structures, confirming specificity [61].

### 2.6. Quantification Using the Optical Fractionator Method

The optical fractionator method represents a precise stereological approach for quantifying cellular populations, amalgamating the functionalities of an optical dissector and a fractionator. Its efficacy in assessing cell numbers across diverse brain regions has been well documented in numerous studies [62,63,64]. Notably, the optical fractionator remains unaffected by histological alterations, shrinkage, or injury-induced expansion [65]. Our aim was to investigate the hypothesis that aging and a sedentary lifestyle exacerbate microglial alterations associated with variations in litter size.

In the histological sections, we precisely identified the layers in the dentate gyrus by positioning counting probes and capturing digital images. This was carried out using a low-magnification 4× objective lens on a NIKON Eclipse 80i microscope (Nikon, Tokyo, Japan), which had a motorized stage (MAC200, Ludl Electronic Products, Hawthorne, NY, USA). The microscope was connected to a computer running the StereoInvestigator software (MicroBrightField, Williston, VT, USA, https://www.mbfbioscience.com/products/stereo-investigator, accessed on 10 April 2024), allowing for the digital recording and analysis of the x, y, and z coordinates of the selected points.

To ensure the accurate detection of microglia using the dissector probe, we replaced the low-resolution objective with a high-resolution 100× oil immersion plan fluoride objective (Nikon, NA 1.3, DF = 0.19 µm). This adjustment enabled the unambiguous identification and counting of microglia.

The thickness of each section was meticulously assessed at every counting site using the high-resolution objective, enabling the precise delineation of the immediate layers at the section’s top and bottom. Considering the variability in thickness and cell distribution across sections, the total count of objects of interest was adjusted based on section thickness. All microglial cell bodies clearly visible within the counting frame were meticulously counted and added to the total marker count. This encompassed cell bodies entirely within the counting frame or intersecting the acceptance line without contacting the rejection line, adhering to criteria established elsewhere [66]. Counting boxes were consistently positioned within a grid in a randomized yet systematic manner to ensure comprehensive coverage and unbiased sampling.

### 2.7. Volume Estimations of Dentate Gyrus Using Planimetric Techniques

Planimetric assessments were performed utilizing the Stereo Investigator software to gauge the volumes of the unilateral dentate gyrus across all experimental conditions. Leveraging the optical fractionator method as the foundational approach for volume calculation, the software ensured consistent section-to-section distances throughout the sequence.

Area estimates from multiple sections were amalgamated to derive a comprehensive estimate of the total volume while also computing the coefficient of error (CE). This methodology proved crucial in estimating volumes based on the actual thickness of histologically prepared sections. Given that shrinkage induced by these processes typically exhibits nonlinearity and predominantly affects the z-axis due to dehydration, all volume estimations were conducted without adjustments for shrinkage.

### 2.8. Photomicrography and Processing

Digital photomicrographs were acquired using a digital camera (MicroFire, Optronics, Goleta, CA, USA) connected to a NIKON Eclipse 80i microscope (Nikon, Tokyo, Japan). Adobe Photoshop software (https://www.adobe.com/sg/products/photoshop.html, accessed on 10 April 2024) was employed for post-capture processing, encompassing adjustments such as scaling and fine-tuning brightness and contrast levels across the entire image.

From the array of micrographs obtained, specific sections from each experimental group were meticulously selected. These selections were based on their proximity to the mean value of microglia number within the region of interest, ensuring that the chosen micrographs offered a representative depiction.

### 2.9. Statistical Analyses

Data are presented as the mean ± standard error of the mean. Supplementary Tables S2–S6 provide detailed summaries of the experimental parameters and average counting results obtained from the optical fractionator. Our grid size was meticulously tailored to achieve an acceptable coefficient of error (CE). To evaluate the CE of total microglial counts per rat, we employed the one-stage systematic sampling procedure (Scheaffer CE), a method utilized in previous studies [67].

The acceptable level of CE for stereological estimations was determined as the ratio between the inherent error introduced by the methodology and the coefficient of variation (CV) [67,68]. For this study, a CE ≤ 0.05 was considered appropriate, as the variance introduced by the estimation procedure minimally contributed to the observed group variance [68]. While the ratio between CE2/CV2 ideally should not exceed 0.5, certain exceptions were identified in our investigation. In these cases, despite higher CE2/CV2 values than recommended, the biological variance and methodology-induced CE were exceedingly low, rendering strict adherence to this rule neither meaningful nor practical [68].

A multifactorial analysis of variance (ANOVA) was performed using ezANOVA v.0.985, a freely available statistical software, to compare stereological estimations among all groups. This was applied as Design 3 Between Subject Factors, followed by pairwise comparisons employing Tukey’s honestly significant difference test (HSD). This test aims to control for multiple comparisons while expressing a standardized Q score. Significance levels were set at *p* < 0.05 to determine statistical significance across the comparisons.

## 3. Results

### 3.1. Body Weights and Dentate Gyrus Volumes

**Figure 3:**
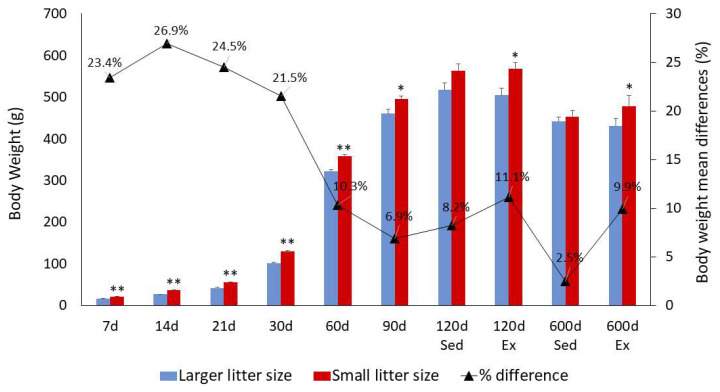
Changes in body weight over time. This figure displays the mean body weight (±standard error of the mean) of rats raised in litters of 6 (small litters) and 12 (large litters), measured at different ages and under various exercise conditions. Statistical significance is indicated by asterisks: (*) denotes significant differences detected by a two-tailed *t*-test, covering the period from the 7th to the 90th postnatal day (PND). For the measurement taken at the 600th PND, significant differences were determined using a three-way ANOVA. The significance levels are as follows: (*) represents *p* < 0.05; (**) represents *p* < 0.01. These results highlight how body weight varies in rats depending on litter size and exercise over time.

Figure 3 provides a comprehensive overview of mean body weight values alongside their respective standard errors across multiple postnatal days (7, 14, 21, 30, 60, 90, 120, and 600). A progressive and significant increase in body weight, irrespective of litter size, was observed until reaching maturity, followed by a decline during aging. Notably, animals from larger litters exhibited significantly lower mean body weights than those from smaller litters between the 7th and 90th postnatal days. However, beyond this period, the mean body weights of rats from both large and small litters became comparable during both the mature (120 days) and aged (600 days) stages, particularly among the sedentary groups. Interestingly, exercised rats from smaller litters displayed higher body weights during these mature and aged periods when compared to exercised rats from larger litters.

Regarding the dentate gyrus volumes, while mature exercised rats from smaller litters showcased higher values and aged sedentary rats from larger litters exhibited smaller volumes on average, no statistically significant differences were detected among the experimental groups. An exception was noted in the aged rats from smaller litters raised sedentarily, who displayed a significant reduction compared to age-matched exercised animals raised in similar-sized litters (F1,32 = 6.16; *p* < 0.018, three-way ANOVA, pairwise comparisons [Q = Tukey HSD: t(8) = 2.40, *p* < 0.0434]).

### 3.2. Impact of Litter Size on Maternal Care

Observations of dams tending to large and small litters unveiled a distinct variance in maternal care, contingent upon litter size. Our investigation primarily focused on behaviors crucially identified by Meaney and colleagues as discriminators between high-care and low-care mothers, specifically, licking/grooming behavior (LG) and arched-back nursing behavior (ABN) [69].

**Figure 4:**
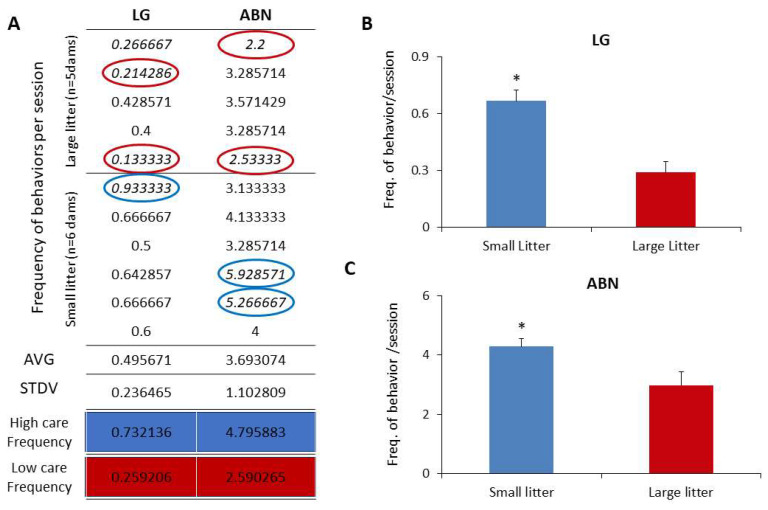
Frequency of maternal behaviors exhibited by dams. (**A**): Average frequency of licking/grooming (LG) and arched-back nursing (ABN) per dam from small and large litters. Dams are categorized as high care (blue) or low care (red) based on their behavior frequency compared to the overall average ± standard deviation. Blue and red circles highlight high care and low care mothers, respectively, detected for each behavior. (**B**,**C**): Comparison of average LG and ABN behaviors among groups of dams from small and large litters. * *t*-test, *p* < 0.05.

Figure 4 illustrates the average occurrences of LG and ABN behaviors per session per dam, complemented by the population mean and standard deviation. Mothers exhibiting high and low care were categorized based on LG and ABN scores ± 1 standard deviation above (high) or below (low) the mean for the entire group. The distribution of licking behavior across the cohort displayed a normal pattern, with dams classified as high and low caregivers predominantly belonging to the small and large litter groups, respectively.

Statistical analysis revealed a significant difference in the averages of LG and ABN behaviors between large litters (0.28 ± 0.12 LG and 2.97 ± 0.57 ABN) and small litters (0.66 ± 0.14 LG and 4.29 ± 1.1 ABN) (*t*-test, *p* < 0.05). Additionally, the frequency of pups being out of the nest was notably higher in large litters compared to small litters.

### 3.3. Cognitive Assays

**Figure 5:**
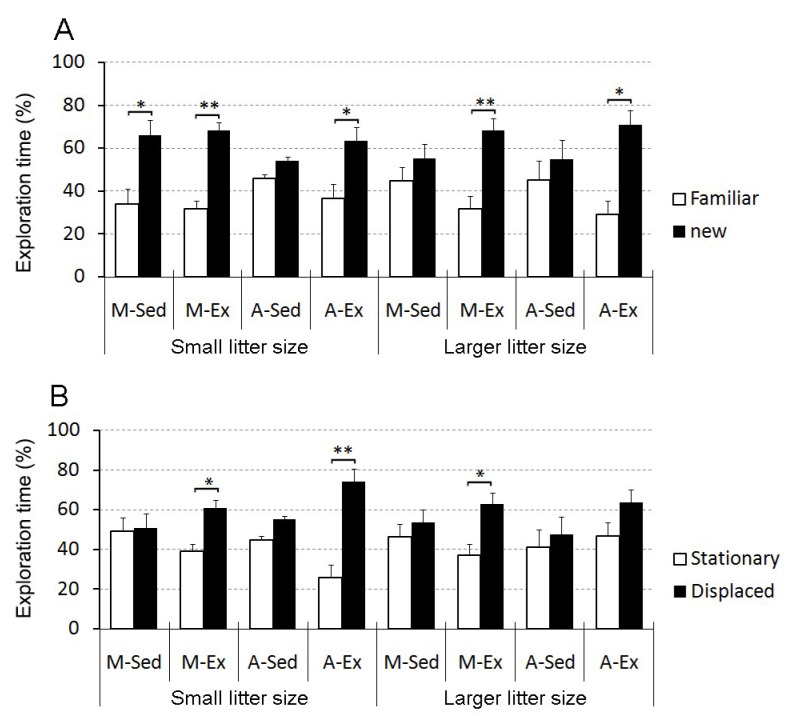
Object recognition and placement analysis. (**A**) Object identity recognition: This section illustrates the results of testing how well different groups of rats recognize specific objects. (**B**) Object placement recognition: This section shows the outcomes of experiments assessing the ability of the same groups of rats to recognize changes in object placement. The groups are defined as follows: M-Sed: mature sedentary rats; M-Ex: mature exercised rats; A-Sed: aged sedentary rats; A-Ex: aged exercised rats. Statistical significance levels are indicated by asterisks: (*) represents a *p*-value of less than 0.05; (**) represents a *p*-value of less than 0.01; the results are based on two-tailed *t*-tests for related samples, illustrating the differences in performance across the experimental groups.

Figure 5 displays the outcomes of the object recognition and spatial memory tests.

Our findings underscore the detrimental impact of a sedentary lifestyle on spatial memory, specifically in both young and aged rats, independent of litter size. However, the exercise proved effective in counteracting these effects among aged rats from smaller litters but did not yield the same benefits in those from larger litters. Conversely, only sedentary–aged rats from both larger and smaller litters display impaired identity object recognition memory, a deficit that was ameliorated by exercise regardless of litter size.

In terms of one-trial object identity recognition, sedentary rats from larger litters, regardless of their age, faced challenges in object discrimination. Yet, exercise emerged as a significant contributor to enhancing their object discrimination abilities, underscoring its consistent impact in mitigating the repercussions of larger litter size, irrespective of the rats’ ages (Figure 5A).

Concerning one-trial object placement recognition, all sedentary rats struggled to distinguish stationary from displaced objects regardless of litter size and age. Conversely, exercised rats from the mature and aged groups from small litters, as well as exercised mature (but not aged) animals, could differentiate between stationary and displaced objects (Figure 5B).

### 3.4. Enhanced Microglial Dynamics in the Dentate Gyrus: Insights from Litter Size, Aging, and Exercise

**Figure 6:**
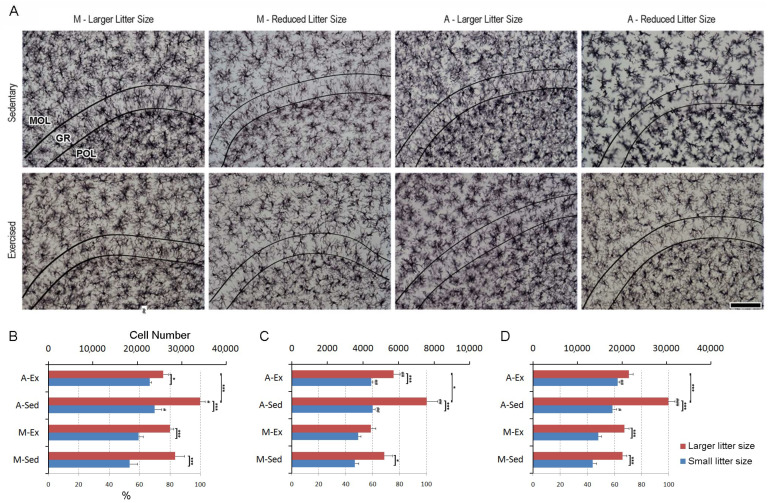
Laminar distribution of microglia in the dentate gyrus of Wistar rats. (**A**) Photomicrographs of immunolabeled sections from the dentate gyrus of mature (M) and aged (A) Wistar rats raised in either small (6 pups per dam) or large litters (12 pups per dam). These rats were subjected to a brief period of exercise (Ex) later in life or remained sedentary (Sed). The images show examples from rats with microglia counts close to the mean for each experimental group. The curved lines indicate the boundaries of the granular layer (GR), which lies between the molecular (MOL) and polymorphic (POL) layers. (**B**–**D**) The graphs display the mean microglial counts, with standard error bars, in the molecular (**B**), granular (**C**), and polymorphic (**D**) layers of the unilateral dentate gyrus. Significance levels are indicated as follows: (#, ## and ###) denote *p* < 0.05, *p* < 0.01 and *p* < 0.001, respectively, when comparing to mature rats; (*) indicates *p* < 0.05; (***) indicates *p* < 0.001. These significance levels represent outcomes from a three-way ANOVA.

The influence of litter size, aging, and exercise on microglial distribution in the dentate gyrus is evident from Figure 6. Figure 6A presents IBA-1-immunolabeled sections highlighting distinct layers under various experimental conditions. The heightened microglial density within the molecular layer among animals from larger litters is particularly striking. Additionally, sedentary older individuals exhibit a significant increase in microglial presence compared to their younger counterparts. Consistent with our hypothesis, exercise interventions seem to attenuate age-related microgliosis, suggesting a potential role of physical activity in reducing heightened microglial activity associated with aging, as depicted in Figure 6B. No notable elevation in microglial numbers can be observed in the granular layer among exercised mature adults from larger litter groups. However, aging manifests in increased microglial counts compared to younger controls. Exercise consistently diminishes microgliosis in aged animals (see Figure 6C).

Similarly, an increase in microglial numbers can be observed across all large litter groups in the granular layer. However, a noteworthy exception is noted in the exercised older group, where a reduction in this effect is evident. Additionally, older groups from smaller litters exhibit an increase in microglial numbers compared to younger groups, particularly in this layer (see Figure 6D). Detailed stereological data can be found in the Supplementary Materials (Tables S1–S6).

The three-way analysis of variance (ANOVA) of the molecular layer indicated significant effects for age (F_(1,32)_ = 10.5, *p* < 0.002768), litter size (F_(1,32)_ = 66.4, *p* < 0.000001), and exercise (F_(1,32)_ = 4.71, *p* < 0.037431). Notably, there were significant interactions between age and exercise (F_(1,32)_ = 8.81, *p* < 0.005622), as well as between litter size and exercise (F_(1,32)_ = 8.82, *p* < 0.005614).

Similarly, the granular layer showed significant effects for age (F_(1,32)_ = 35.5, *p* < 0.000001), litter size (F_(1,32)_ = 50.9, *p* < 0.000001), and exercise (F_(1,32)_ = 7.26, *p* < 0.011118). Additionally, this layer exhibited significant interactions between age and litter size (F_(1,32)_ = 4.58, *p* < 0.040107), as well as between litter size and exercise (F_(1,32)_ = 8.54, *p* < 0.006321).

In the polymorphic layer, the results showed significant effects for age (F_(1,32_) = 57.3, *p* < 0.000001), litter size (F(_1,32_) = 104, *p* < 0.000001), and exercise (F(_1,32)_ = 4.91, *p* < 0.033878). This layer also revealed significant interactions between age and exercise (F_(1,32)_ = 12.3, *p* < 0.001351), litter size and exercise (F_(1,32)_ = 16.4, *p* < 0.000306), and among all three variables (F_(1,32)_ = 12.3, *p* < 0.001392).

**Figure 7:**
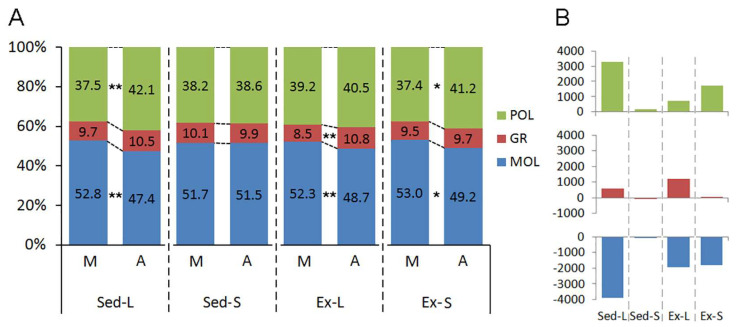
Laminar redistribution of microglia in the rat dentate gyrus after early-life litter size changes and exercise. (**A**) This panel shows the percentage distribution of microglial counts within the polymorphic (POL), granular (GR), and molecular (MOL) layers of the rat dentate gyrus across various experimental conditions. This comparison assesses how early-life litter size changes and subsequent exercise impact microglial distribution. (**B**) This panel presents the absolute numbers of microglia in each layer, with the polymorphic layer at the top, the granular layer in the middle, and the molecular layer at the bottom. The data illustrate how microglial counts differ among groups with varying litter sizes and exercise conditions. The following abbreviations are used for the experimental groups: M: mature adult rats; A: aged rats; Sed-L: sedentary rats from large litters; Sed-S: sedentary rats from small litters; Ex-L: exercised rats from large litters; Ex-S: exercised rats from small litters. The asterisks indicate levels of statistical significance as follows: (*) denotes *p* < 0.05; (**) denotes *p* < 0.01. Significances were detected by 3-way ANOVA.

Figure 7 delineates the impact of litter size, aging, and exercise on the laminar distribution of microglia within the dentate gyrus of both mature and aged rats. Notable laminar redistributions of microglia are associated primarily with litter size and aging, while exercise interventions appear to mitigate these laminar alterations.

Aging does not alter the laminar distribution of microglia in the Sed-S groups. However, perinatal changes linked to increased litter size led to a significant shift in cell distribution, particularly elevating the proportion of cells in the granular layer compared to the molecular layer (Sed-L). Exercise interventions further accentuate this shift by increasing the proportion of microglia in the granular layer (Ex-L). Furthermore, albeit to a lesser extent, aging within the Ex-S groups also exhibits a higher proportion of microglia in the polymorphic layer compared to the molecular layer.

It is important to highlight that while these findings illuminate intriguing dynamics of microglial populations, no direct correlations were identified between the quantity of microglia and behavioral performance.

## 4. Discussion

We investigated the effects of litter size and late-life treadmill exercise on the laminar distribution of microglia in the dentate gyrus of mature and aged rats. Our findings revealed that exercise and a decrease in litter size reduced the number of microglia. Notably, this reduction correlated with preserving spatial and object recognition memories. Interestingly, compared to exercised animals of the same age from small litters, sedentary rats from larger litters exhibited a subtle yet enduring decline in dentate gyrus volume.

### 4.1. Litter Size, Growth, and Somatic Maturation

Our experimental approach drew inspiration from a protocol outlined elsewhere [70], with a modification to limit the number of pups within the larger litters to 12 per dam, aiming to prevent undernourishment. In our model, pups from different dams were combined and redistributed among the dams, resulting in varying ratios of pups per dam. Specifically, the large litter group maintained a pup-to-dam ratio of 12:1, while the small litter group had a ratio of 6:1.

This experimental manipulation was predicated on the assumption that a greater ratio of pups to dam would result in less suckling per pup, a pattern observed in earlier studies. This anticipated reduction in nursing interactions directly influences maternal care behaviors [23,69].

The alterations observed in maternal care and the presumed differences in milk demand affecting the number of breastfeeding per pup align with the body weight curve findings, revealing significant differences from postnatal days 7 to 90. However, it is crucial to note a prior study [71] that identified three classes of natural litter sizes in Wistar rats; they found minor distinctions in pup growth and somatic maturation within these classes: Class 1 (6, 7, and 8 pups), Class 2 (9 and 10 pups), and Class 3 (11 and 12 pups). Our experimental design pertained to Classes 1 (6 pups/dam) and 3 (12 pups/dam).

Moreover, the existing literature has demonstrated that the typical litter size for Wistar rats ranges from 1 to 13 pups, and no undernutrition occurs with a ratio of 6 or 12 pups per dam during the weaning period [71,72].

Taking these findings together, it is plausible to hypothesize that the observed behavioral and microglial alterations were triggered by changes associated with modified maternal care rather than variations in the subjects’ nutritional status.

It is important to emphasize, however, that relying solely on body weight may not fully capture a complete picture of physical composition and health, especially considering how exercise can affect the balance between fat and lean mass [73,74], as well as its impact on hormonal levels [75,76]. Recognizing these factors is essential for improving accuracy in future studies. Simple measurements like body mass index (BMI) values and fat percentage calculations can significantly enhance our understanding of the metabolic effects of exercise and early-life nutritional interventions in animals [77].

### 4.2. Litter Size and Microglial Response

The impact of litter size on the microglial response has captivated research interest, particularly within investigations into the epigenetic effects of maternal behavior during early-life experiences. Pioneering studies by Michael Meaney and colleagues underscored how maternal behavior—specifically, grooming and licking—profoundly influences genes governing the hypothalamic–pituitary–adrenal axis [78]. Their research illuminated the far-reaching epigenetic effects of early maternal care on these genetic mechanisms. Considering the pivotal role of glucocorticoids in shaping microglial function across the lifespan [79,80] and prior indications linking litter size with corticosterone levels [18], it is reasonable to anticipate that changes in microglial responses due to variations in litter size may be associated with epigenetic effects of altered maternal care. Substantiating this expectation, studies conducting an optical density analysis of IBA-1’s immunoreactivity in the dentate gyrus uncovered significant differences between adult and aged groups subjected to chronic restraint stress compared to control groups. These differences were associated with heightened corticosterone levels in the stressed adult and aged groups [81]. This suggests that diminished maternal care during early life might disrupt the innate immune response in adulthood, potentially leaving lasting imprints on the offspring’s immune system [8]. Further studies including the analysis of DNA methylation and glucocorticoid levels with microglial profile analysis may shed light on the possible mechanisms involved in the effects described herein and possibly confirm these speculations.

In our study, manipulating the pup-to-dam ratio resulted in discernible differences in maternal care status. Notably, pups in the high-ratio group received less maternal grooming and licking than those in the low-ratio group. The distribution of maternal behaviors followed a normal, non-bimodal pattern, with high- and low-care mothers existing as extremes on a continuum, as observed previously [1]. By categorizing females exhibiting licking behavior one standard deviation below or above the mean, we could compare groups that experienced a 2- to 3-fold difference in received maternal care without accounting for the division of care due to the number of puppies. Studies with varying litter sizes have indicated that larger litters consistently receive reduced individual care per pup [82].

Litter size, in early life, has the potential to significantly impact the social interactions among offspring, potentially leading to enduring alterations in anxiety levels, responses to new experiences, and the ability to cope with stress in adulthood [83]. However, the precise contribution of reduced maternal care versus interactions among offspring to cognitive and emotional changes later in life and their potential interaction remains to be precisely quantified.

However, information on the long-term consequences of litter size during brain development on microglia later in life is lacking. Our findings demonstrate a lasting impact on microglial numbers in the dentate gyrus of young and aged rats from larger litters during the suckling period. A notable increase in microglial numbers was observed in sedentary aged rats raised in large litters, and it was suggested that ad libitum access to food and water combined with late-life exercise did not suffice to reverse these changes. In larger litters, brain development is associated with enduring alterations in the brain’s innate immune system, significantly impacting microglial homeostasis in aged rats. Whether a direct correlation exists between litter size, age-related plasticity in the dentate gyrus, and changes in microglial numbers remains to be established.

Moreover, we observed a laminar redistribution of microglia associated with litter size and aging, apparently mitigated by exercise. It is well known that the laminar organization of dentate gyrus plays a crucial role in processing and integrating incoming neural signals. Microglia, in this context, are strategically distributed across these layers, suggesting a potential role in modulating synaptic transmission and plasticity [84]. Changes in the laminar distribution of microglia may suggest a spatially specific response to various stimuli, which could influence synaptic plasticity and cognitive functions differently across layers [85].

The dentate gyrus is also known for its role in adult neurogenesis and synaptic plasticity, both of which are fundamental for learning and memory processes and known to be influenced by exercise and early environmental conditions. It has been demonstrated that cellular differentiation and the subregional distribution of microglia follow specific developmental gradients of the different parts of Ammon’s horn and the dentate gyrus [86]. Given microglia’s established role in modulating neuronal activity and synaptic connectivity through the secretion of various factors, it stands to reason that alterations in their laminar distribution may contribute to the region-specific regulation of neurogenesis and synaptic remodeling within the dentate gyrus, thereby influencing cognitive functions. Thus, as previously noted, a comprehensive characterization of microglial profiles across this distribution is essential to discern whether the observed laminar reorganization exerts a protective or detrimental effect mediated by microglia. While morphological classification could have provided valuable insights, it is important to acknowledge that we did not undertake such classification in this study, representing a limitation that warrants consideration in future investigations.

### 4.3. Litter Size, Aging, and Cognitive Decline

Under normal conditions, microglia in various CNS regions exhibit distinct branching and ramified structures, setting them apart from tissue macrophages [87]. Despite the absence of neurological diseases, aging prompts a shift towards more reactive forms of astrocytes and microglia, contributing to an increased and sustained pro-inflammatory profile [88,89]. Age-related changes in microglia involve altered cytokine production [90], modified activation marker expression [91], and morphological changes characterized by dystrophy [92]. Furthermore, during aging, the bidirectional communication between neurons and microglia may become disrupted, leading to the loss of neuronal-derived factors that typically regulate microglial activation [93].

It has been reported that while a significant rise in pro-inflammatory microglial profiles is observed in the hippocampus and dentate gyrus during aging, this does not necessarily correlate directly with cognitive decline [94]. Additionally, a higher number of these profiles is identified in the dentate gyrus of sedentary animals [24,95], making it challenging to directly link microglial changes with memory impairments. However, exercise appears to curb microglial proliferation and enhance the expression of a pro-neurogenic phenotype specifically in the hippocampus and dentate gyrus [24,95].

In alignment with these findings, our study did not detect cognitive impairments in the exercised group except in aged animals from larger litters, for which exercise failed to mitigate spatial memory decline. Notably, this group displayed a higher number of microglia in all layers of the dentate gyrus compared to age-matched exercised rats. Our observations suggest a potential presence of two distinct microglial phenotypes (pro-neurogenic and pro-inflammatory ones) within the dentate gyrus of Wistar rats. Furthermore, it is plausible that exercised rats primarily exhibit the dominant effects of the pro-neurogenic phenotype, as previously described in mice [24,95]. Nevertheless, it is important to note that, in our work we have assessed the total number and laminar distribution of microglial cells in DG of the experimental subjects, with no further assessment of the molecular profile of these cells; therefore, we can only suppose, based on the abovementioned observations, that the changes reported here may be related to a rise in pro-inflammatory profiles induced by aging, reduced maternal care, and a sedentary lifestyle, leading to changes in cognitive functions. However, whether the increased numbers of microglia in fact translate into increased inflammation patterns or reflect a protective mechanism against detrimental environmental conditions is a question that demands further investigation.

### 4.4. Technical Limitations

Estimating object numbers in histological sections using stereological methods often exhibit variability across studies due to distinct estimation techniques, animal lineages, histological procedures, stereological protocols, and even uncertainties in defining objects and areas of interest [96]. We standardized our procedures across all samples to mitigate potential errors when comparing animal groups. We maintained uniformity by employing identical protocols for sample processing, data collection, and analysis through the consistent use of stereological methods, software, and hardware.

Furthermore, we implemented rigorous verification procedures to ascertain consistency in identifying the objects of interest. Multiple investigators independently counted the same regions using the identical anti-Iba1 antibody as a microglial marker. This meticulous approach aimed to minimize potential variations stemming from non-biological sources, ensuring reliability in our data interpretation.

Additionally, it is worth noting that corticosteroids, which can inhibit microglial activation, can influence microglial plasticity [97]. During our experiments, treadmill exercise may have induced stress, potentially altering plasma corticosteroid levels and, consequently, impacting microglial numbers. Although we did not measure plasma corticosteroid levels post-exercise, it is a factor that warrants consideration, as it might contribute to the observations made in our study.

Moreover, the behavioral testing protocol may have induced a bias in the cognitive results. Pre-exposure of animals to cognitive tests when mature followed by re-exposure to the same test when aged can potentially influence the results due to several behavioral factors, such as habituation effects, that may enhance performance during retesting, confounding interpretations of age-related cognitive changes. Nevertheless, considering the fact that all experimental groups underwent the same protocols under the same conditions, these effects may not have influenced the general outcomes of this study. Additionally, limiting the analysis to a subset of cognitive parameters may not have allowed the study to capture the full spectrum of cognitive changes induced by the experimental conditions.

The absence of sex comparisons represents another limitation of this study. Sex differences in cognitive function have been widely documented in both human and animal research, with evidence suggesting that males and females may exhibit distinct patterns of cognitive performance and response to environmental factors. By exclusively using male rats as experimental subjects, this study overlooks potential sex-specific effects that could contribute to variations in cognitive outcomes. Incorporating both male and female subjects and conducting sex-specific analyses would allow for a more nuanced understanding of how maternal care, aging, and exercise interact with biological sex to influence cognitive function. Additionally, such analyses could shed light on potential sex-specific vulnerabilities or protective factors that may have relevance for translational research in humans.

## 5. Conclusions

This study explores the multifaceted influence of litter size, maternal care, exercise, and aging on neurobehavioral plasticity and dentate gyrus microglia dynamics in rats. Body weight evolution revealed a progressive increase until maturity, followed by a decline during aging, with larger-litter rats exhibiting lower weights initially. Notably, exercised rats from smaller litters displayed higher body weights during the mature and aged stages. The dentate gyrus volumes showed no significant differences among the groups, except for the aged sedentary rats from smaller litters, who exhibited a reduction. Maternal care varied significantly based on litter size, with large litter dams showing lower frequencies of caregiving behaviors. Behavioral assays highlighted the detrimental impact of a sedentary lifestyle on spatial memory, mitigated by exercise in aged rats from smaller litters. Microglial dynamics in the dentate gyrus revealed age-related changes, modulated by litter size and exercise. Exercise interventions mitigated microgliosis associated with aging, particularly in aged rats. These findings underscore the complex interplay between early-life experiences, exercise, and microglial dynamics in neurobehavioral outcomes during aging.

## Figures and Tables

**Table 1:** Parameters of physical exercise (treadmill running).

Weeks	1st	2nd	3rd	4th	5th
Duration of the daily sessions	30 min	30 min	30 min	45 min	45 min
Number of sessions per week	5	5	5	3	2
Running speed	5 m/min	10 m/min	15 m/min	25 m/min	25 m/min

## Data Availability

The article and its Supplementary Materials provide the primary data underlying this study. Additional inquiries regarding data availability can be addressed to the corresponding author. Tabulated data about behavioral data, body weight, maternal care, granular, molecular, and polymorphic layers can be accessed at: https://doi.org/10.20944/preprints202404.0898.v1, accessed on 1 May 2024.

## Supplementary Materials

The following supporting information can be downloaded at: https://www.mdpi.com/article/10.3390/brainsci14050497/s1, Table S1. Microglial granular layer estimates for aged, exercised and sedentary rats raised in large and small litters. Experimental parameters, optical fractionator counting results and individual unilateral microglial numbers (N) and mean groups with the coefficient of error (CE). Table S2. Microglial granular layer estimates for mature, exercised and sedentary rats raised in large and small litters. Experimental parameters, optical fractionator counting results and individual unilateral microglial numbers (N) and mean groups with the coefficient of error (CE). Table S3. Microglial molecular layer estimates for aged, exercised and sedentary subjects raised in large and small litters. Experimental parameters, optical fractionator counting results and individual unilateral microglial numbers (N) and mean groups with the coefficient of error (CE). Table S4. Microglial molecular layer estimates for mature, exercised and sedentary rats raised in large and small litters. Experimental parameters, optical fractionator counting results and individual unilateral microglial numbers (N) and mean groups with the coefficient of error (CE). Table S5. Microglial polymorphic layer estimates for aged, exercised and sedentary rats raised in large and small litters. Experimental parameters, optical fractionator counting results and individual unilateral microglial numbers (N) and mean groups with the coefficient of error (CE). Table S6. Microglial polymorphic layer estimates for mature, exercised and sedentary rats raised in large and small litters. Experimental parameters, optical fractionator counting results and individual unilateral microglial numbers (N) and mean groups with the coefficient of error (CE).

## References

[1] Curley J.P., Champagne F.A. (2016). Influence of maternal care on the developing brain: Mechanisms, temporal dynamics and sensitive periods. Front. Neuroendocrinol..

[2] Nishi M. (2020). Effects of Early-Life Stress on the Brain and Behaviors: Implications of Early Maternal Separation in Rodents. Int. J. Mol. Sci..

[3] Čater M., Majdič G. (2022). How early maternal deprivation changes the brain and behavior?. Eur. J. Neurosci..

[4] Hegde A., Mitra R. (2020). Environment and early life: Decisive factors for stress-resilience and vulnerability. Int. Rev. Neurobiol..

[5] Kundakovic M., Champagne F.A. (2015). Early-life experience, epigenetics, and the developing brain. Neuropsychopharmacology.

[6] Seay B., Hansen E., Harlow H.F. (1962). Mother-infant separation in monkeys. J. Child Psychol. Psychiatry.

[7] Suchecki D. (2018). Maternal regulation of the infant’s hypothalamic-pituitary-adrenal axis stress response: Seymour ‘Gig’ Levine’s legacy to neuroendocrinology. J. Neuroendocrinol..

[8] Eid R.S., Chaiton J.A., Lieblich S.E., Bodnar T.S., Weinberg J., Galea L.A.M. (2019). Early and late effects of maternal experience on hippocampal neurogenesis, microglia, and the circulating cytokine milieu. Neurobiol. Aging.

[9] Hanamsagar R., Alter M.D., Block C.S., Sullivan H., Bolton J.L., Bilbo S.D. (2017). Generation of a microglial developmental index in mice and in humans reveals a sex difference in maturation and immune reactivity. Glia.

[10] Roque A., Ochoa-Zarzosa A., Torner L. (2016). Maternal separation activates microglial cells and induces an inflammatory response in the hippocampus of male rat pups, independently of hypothalamic and peripheral cytokine levels. Brain Behav. Immun..

[11] Bachiller S., Paulus A., Vázquez-Reyes S., García-Domínguez I., Deierborg T. (2020). Maternal separation leads to regional hippocampal microglial activation and alters the behavior in the adolescence in a sex-specific manner. Brain Behav. Immun. Health.

[12] Reshetnikov V., Ryabushkina Y., Kovner A., Lepeshko A., Bondar N. (2020). Repeated and single maternal separation specifically alter microglial morphology in the prefrontal cortex and neurogenesis in the hippocampus of 15-day-old male mice. NeuroReport.

[13] Banqueri M., Méndez M., Gómez-Lázaro E., Arias J.L. (2019). Early life stress by repeated maternal separation induces long-term neuroinflammatory response in glial cells of male rats. Stress.

[14] Nicolas S., McGovern A.J., Hueston C.M., O’Mahony S.M., Cryan J.F., O’Leary O.F., Nolan Y.M. (2022). Prior maternal separation stress alters the dendritic complexity of new hippocampal neurons and neuroinflammation in response to an inflammatory stressor in juvenile female rats. Brain Behav. Immun..

[15] Hou M., Liu Y., Zhu L., Sun B., Guo M., Burén J., Li X. (2011). Neonatal overfeeding induced by small litter rearing causes altered glucocorticoid metabolism in rats. PLoS ONE.

[16] Carvalho A.L.O., Ferri B.G., de Sousa F.A.L., Vilela F.C., Giusti-Paiva A. (2016). Early life overnutrition induced by litter size manipulation decreases social play behavior in adolescent male rats. Int. J. Dev. Neurosci..

[17] Rodrigues A.L., de Moura E.G., Passos M.C., Dutra S.C., Lisboa P.C. (2009). Postnatal early overnutrition changes the leptin signalling pathway in the hypothalamic-pituitary-thyroid axis of young and adult rats. J. Physiol..

[18] Rodel H.G., Meyer S., Prager G., Stefanski V., Hudson R. (2010). Litter size is negatively correlated with corticosterone levels in weanling and juvenile laboratory rats. Physiol. Behav..

[19] Velkoska E., Cole T.J., Dean R.G., Burrell L.M., Morris M.J. (2008). Early undernutrition leads to long-lasting reductions in body weight and adiposity whereas increased intake increases cardiac fibrosis in male rats. J. Nutr..

[20] Davidowa H., Li Y., Plagemann A. (2002). Hypothalamic ventromedial and arcuate neurons of normal and postnatally overnourished rats differ in their responses to melanin-concentrating hormone. Regul. Pept..

[21] Prager G., Stefanski V., Hudson R., Rodel H.G. (2010). Family matters: Maternal and litter-size effects on immune parameters in young laboratory rats. Brain Behav. Immun..

[22] Enes-Marques S., Giusti-Paiva A. (2018). Litter size reduction accentuates maternal care and alters behavioral and physiological phenotypes in rat adult offspring. J. Physiol. Sci..

[23] Cortes-Barberena E., Gonzalez-Marquez H., Gomez-Olivares J.L., Ortiz-Muniz R. (2008). Effects of moderate and severe malnutrition in rats on splenic T lymphocyte subsets and activation assessed by flow cytometry. Clin. Exp. Immunol..

[24] Kohman R.A., DeYoung E.K., Bhattacharya T.K., Peterson L.N., Rhodes J.S. (2012). Wheel running attenuates microglia proliferation and increases expression of a proneurogenic phenotype in the hippocampus of aged mice. Brain Behav. Immun..

[25] Hudson R., Maqueda B., Velazquez Moctezuma J., Morales Miranda A., Rodel H.G. (2011). Individual differences in testosterone and corticosterone levels in relation to early postnatal development in the rabbit Oryctolagus cuniculus. Physiol. Behav..

[26] Ziko I., De Luca S., Dinan T., Barwood J.M., Sominsky L., Cai G., Kenny R., Stokes L., Jenkins T.A., Spencer S.J. (2014). Neonatal overfeeding alters hypothalamic microglial profiles and central responses to immune challenge long-term. Brain Behav. Immun..

[27] De Luca S.N., Ziko I., Dhuna K., Sominsky L., Tolcos M., Stokes L., Spencer S.J. (2017). Neonatal overfeeding by small-litter rearing sensitises hippocampal microglial responses to immune challenge: Reversal with neonatal repeated injections of saline or minocycline. J. Neuroendocrinol..

[28] Tapia-Gonzalez S., Garcia-Segura L.M., Tena-Sempere M., Frago L.M., Castellano J.M., Fuente-Martin E., Garcia-Caceres C., Argente J., Chowen J.A. (2011). Activation of microglia in specific hypothalamic nuclei and the cerebellum of adult rats exposed to neonatal overnutrition. J. Neuroendocrinol..

[29] Viana L.C., Lima C.M., Oliveira M.A., Borges R.P., Cardoso T.T., Almeida I.N.F., Diniz D.G., Bento-Torres J., Pereira A., Batista-de-Oliveira M. (2013). Litter size, age-related memory impairments, and microglial changes in rat dentate gyrus: Stereological analysis and three dimensional morphometry. Neuroscience.

[30] Bredy T.W., Humpartzoomian R.A., Cain D.P., Meaney M.J. (2003). Partial reversal of the effect of maternal care on cognitive function through environmental enrichment. Neuroscience.

[31] Bredy T.W., Zhang T.Y., Grant R.J., Diorio J., Meaney M.J. (2004). Peripubertal environmental enrichment reverses the effects of maternal care on hippocampal development and glutamate receptor subunit expression. Eur. J. Neurosci..

[32] Baroncelli L., Braschi C., Spolidoro M., Begenisic T., Sale A., Maffei L. (2010). Nurturing brain plasticity: Impact of environmental enrichment. Cell Death Differ..

[33] Xiao K., Luo Y., Liang X., Tang J., Wang J., Xiao Q., Qi Y., Li Y., Zhu P., Yang H. (2021). Beneficial effects of running exercise on hippocampal microglia and neuroinflammation in chronic unpredictable stress-induced depression model rats. Transl. Psychiatry.

[34] Littlefield A.M., Setti S.E., Priester C., Kohman R.A. (2015). Voluntary exercise attenuates LPS-induced reductions in neurogenesis and increases microglia expression of a proneurogenic phenotype in aged mice. J. Neuroinflamm..

[35] Moita L., Lustosa M.F., Silva A.T., Pires-de-Melo I.H., de Melo R.J., de Castro R.M., Filho N.T., Ferraz J.C., Leandro C.G. (2011). Moderate physical training attenuates the effects of perinatal undernutrition on the morphometry of the splenic lymphoid follicles in endotoxemic adult rats. Neuroimmunomodulation.

[36] Delprato A., Bonheur B., Algéo M.P., Rosay P., Lu L., Williams R.W., Crusio W.E. (2015). Systems genetic analysis of hippocampal neuroanatomy and spatial learning in mice. Genes. Brain Behav..

[37] Crusio W.E., Schwegler H. (2005). Learning spatial orientation tasks in the radial-maze and structural variation in the hippocampus in inbred mice. Behav. Brain Funct..

[38] Di Castro M.A., Volterra A. (2022). Astrocyte control of the entorhinal cortex-dentate gyrus circuit: Relevance to cognitive processing and impairment in pathology. Glia.

[39] Danieli K., Guyon A., Bethus I. (2023). Episodic Memory formation: A review of complex Hippocampus input pathways. Prog. Neuropsychopharmacol. Biol. Psychiatry.

[40] Asim M., Wang H., Chen X. (2024). Shedding light on cholecystokinin’s role in hippocampal neuroplasticity and memory formation. Neurosci. Biobehav. Rev..

[41] Baudry M., Bi X. (2024). Revisiting the calpain hypothesis of learning and memory 40 years later. Front. Mol. Neurosci..

[42] Förster E., Zhao S., Frotscher M. (2006). Laminating the hippocampus. Nat. Rev. Neurosci..

[43] van Groen T., Miettinen P., Kadish I. (2003). The entorhinal cortex of the mouse: Organization of the projection to the hippocampal formation. Hippocampus.

[44] Diniz D.G., de Oliveira M.A., de Lima C.M., Foro C.A.R., Sosthenes M.C.K., Bento-Torres J., Vasconcelos P.F.D., Anthony D.C., Diniz C.W.P. (2016). Age, environment, object recognition and morphological diversity of GFAP-immunolabeled astrocytes. Behav. Brain Funct..

[45] Diniz D.G., Foro C.A.R., Rego C.M.D., Gloria D.A., de Oliveira F.R.R., Paes J.M.P., de Sousa A.A., Tokuhashi T.P., Trindade L.S., Turiel M.C.P. (2010). Environmental impoverishment and aging alter object recognition, spatial learning, and dentate gyrus astrocytes. Eur. J. Neurosci..

[46] Jans J.E., Woodside B. (1987). Effects of litter age, litter size, and ambient temperature on the milk ejection reflex in lactating rats. Dev. Psychobiol..

[47] Morag M., Popliker F., Yagil R. (1975). Effect of litter size on milk yield in the rat. Lab. Anim..

[48] Yagil R., Etzion Z., Berlyne G.M. (1976). Changes in rat milk quantity and quality due to variations in litter size and high ambient temperature. Lab. Anim. Sci..

[49] Caldji C., Tannenbaum B., Sharma S., Francis D., Plotsky P.M., Meaney M.J. (1998). Maternal care during infancy regulates the development of neural systems mediating the expression of fearfulness in the rat. Proc. Natl. Acad. Sci. USA.

[50] Uriarte N., Breigeiron M.K., Benetti F., Rosa X.F., Lucion A.B. (2007). Effects of maternal care on the development, emotionality, and reproductive functions in male and female rats. Dev. Psychobiol..

[51] Brooks G.A., White T.P. (1978). Determination of metabolic and heart rate responses of rats to treadmill exercise. J. Appl. Physiol. Respir. Environ. Exerc. Physiol..

[52] Vanzella C., Neves J.D., Vizuete A.F., Aristimunha D., Kolling J., Longoni A., Gonçalves C.A.S., Wyse A.T.S., Netto C.A. (2017). Treadmill running prevents age-related memory deficit and alters neurotrophic factors and oxidative damage in the hippocampus of Wistar rats. Behav. Brain Res..

[53] Vanzella C., Sanches E.F., Odorcyk F.K., Nicola F., Kolling J., Longoni A., Dos Santos T.M., Wyse A.T.S., Netto C.A. (2017). Forced Treadmill Exercise Prevents Spatial Memory Deficits in Aged Rats Probably Through the Activation of Na. Neurochem. Res..

[54] Ennaceur A., Michalikova S., Bradford A., Ahmed S. (2005). Detailed analysis of the behavior of Lister and Wistar rats in anxiety, object recognition and object location tasks. Behav. Brain Res..

[55] Dere E., Huston J.P., De Souza Silva M.A. (2007). The pharmacology, neuroanatomy and neurogenetics of one-trial object recognition in rodents. Neurosci. Biobehav. Rev..

[56] Tulving E. (2002). Episodic memory: From mind to brain. Annu. Rev. Psychol..

[57] Tulving E. (2001). Episodic memory and common sense: How far apart?. Philos. Trans. R. Soc. Lond. B Biol. Sci..

[58] Dere E., Huston J.P., De Souza Silva M.A. (2005). Episodic-like memory in mice: Simultaneous assessment of object, place and temporal order memory. Brain Res. Brain Res. Protoc..

[59] Dix S.L., Aggleton J.P. (1999). Extending the spontaneous preference test of recognition: Evidence of object-location and object-context recognition. Behav. Brain Res..

[60] Shu S., Ju G., Fan L. (1988). The glucose oxidase-DAB-nickel method in peroxidase histochemistry of the nervous system. Neurosci. Lett..

[61] Saper C.B., Sawchenko P.E. (2003). Magic peptides, magic antibodies: Guidelines for appropriate controls for immunohistochemistry. J. Comp. Neurol..

[62] West M.J. (2002). Design-based stereological methods for counting neurons. Prog. Brain Res..

[63] West M.J. (1999). Stereological methods for estimating the total number of neurons and synapses: Issues of precision and bias. Trends Neurosci..

[64] Bonthius D.J., McKim R., Koele L., Harb H., Karacay B., Mahoney J., Pantazis N.J. (2004). Use of frozen sections to determine neuronal number in the murine hippocampus and neocortex using the optical disector and optical fractionator. Brain Res. Brain Res. Protoc..

[65] West M.J., Slomianka L., Gundersen H.J. (1991). Unbiased stereological estimation of the total number of neurons in thesubdivisions of the rat hippocampus using the optical fractionator. Anat. Rec..

[66] Gundersen H., Jensen E. (1987). The efficiency of systematic sampling in stereology and its prediction. J. Microsc..

[67] Glaser E.M., Wilson P.D. (1998). The coefficient of error of optical fractionator population size estimates: A computer simulation comparing three estimators. J. Microsc..

[68] Slomianka L., West M.J. (2005). Estimators of the precision of stereological estimates: An example based on the CA1 pyramidal cell layer of rats. Neuroscience.

[69] Meaney M.J. (2001). Maternal care, gene expression, and the transmission of individual differences in stress reactivity across generations. Annu. Rev. Neurosci..

[70] Celedon J.M., Santander M., Colombo M. (1979). Long-term effects of early undernutrition and environmental stimulation on learning performance of adult rats. J. Nutr..

[71] Chahoud I., Paumgartten F.J. (2009). Influence of litter size on the postnatal growth of rat pups: Is there a rationale for litter-size standardization in toxicity studies?. Environ. Res..

[72] Bulfin L.J., Clarke M.A., Buller K.M., Spencer S.J. (2011). Anxiety and hypothalamic-pituitary-adrenal axis responses to psychological stress are attenuated in male rats made lean by large litter rearing. Psychoneuroendocrinology.

[73] Lee H.S., Lee J. (2021). Effects of exercise interventions on weight, body mass index, lean body mass and accumulated visceral fat in overweight and obese individuals: A systematic review and meta-analysis of randomized controlled trials. Int. J. Environ. Res. Public Health.

[74] Boschetti D., Muller C.R., Américo A.L.V., Vecchiatto B., Martucci L.F., Pereira R.O., Oliveira C.P., Fiorino P., Evangelista F.S., Azevedo-Martins A.K. (2021). Aerobic Physical Exercise Improves Exercise Tolerance and Fasting Glycemia Independent of Body Weight Change in Obese Females. Front. Endocrinol..

[75] Mani B.K., Castorena C.M., Osborne-Lawrence S., Vijayaraghavan P., Metzger N.P., Elmquist J.K., Zigman J.M. (2018). Ghrelin mediates exercise endurance and the feeding response post-exercise. Mol. Metab..

[76] Hwang E., Portillo B., Grose K., Fujikawa T., Williams K.W. (2023). Exercise-induced hypothalamic neuroplasticity: Implications for energy and glucose metabolism. Mol. Metab..

[77] Remmers F., Fodor M., de Waal H.A.D.-V. (2008). Neonatal food restriction permanently alters rat body dimensions and energy intake. Physiol. Behav..

[78] Meaney M.J., Szyf M. (2005). Environmental programming of stress responses through DNA methylation: Life at the interface between a dynamic environment and a fixed genome. Dialogues Clin. Neurosci..

[79] van Olst L., Bielefeld P., Fitzsimons C.P., de Vries H.E., Schouten M. (2018). Glucocorticoid-mediated modulation of morphological changes associated with aging in microglia. Aging Cell.

[80] Barrientos R.M., Thompson V.M., Kitt M.M., Amat J., Hale M.W., Frank M.G., Crysdale N.Y., Stamper C.E., Hennessey P.A., Watkins L.R. (2015). Greater glucocorticoid receptor activation in hippocampus of aged rats sensitizes microglia. Neurobiol. Aging.

[81] Park J.H., Yoo K.Y., Lee C.H., Kim I.H., Shin B.N., Choi J.H., Hwang I.K., Won M.H. (2011). Comparison of glucocorticoid receptor and ionized calcium-binding adapter molecule 1 immunoreactivity in the adult and aged gerbil hippocampus following repeated restraint stress. Neurochem. Res..

[82] Dimitsantos E., Escorihuela R.M., Fuentes S., Armario A., Nadal R. (2007). Litter size affects emotionality in adult male rats. Physiol. Behav..

[83] Calisir M., Yilmaz O., Kolatan H.E., Sezgin A.K. (2023). Effects of litter size and caging on physical and mental development in rats. Physiol. Behav..

[84] Collazos-Castro J.E., Nieto-Sampedro M. (2001). Developmental and reactive growth of dentate gyrus afferents: Cellular and molecular interactions. Restor. Neurol. Neurosci..

[85] Jinno S., Fleischer F., Eckel S., Schmidt V., Kosaka T. (2007). Spatial arrangement of microglia in the mouse hippocampus: A stereological study in comparison with astrocytes. Glia.

[86] Dalmau I., Finsen B., Zimmer J., González B., Castellano B. (1998). Development of microglia in the postnatal rat hippocampus. Hippocampus.

[87] Ransohoff R.M., Perry V.H. (2009). Microglial physiology: Unique stimuli, specialized responses. Annu. Rev. Immunol..

[88] Davies D.S., Ma J., Jegathees T., Goldsbury C. (2017). Microglia show altered morphology and reduced arborization in human brain during aging and Alzheimer’s disease. Brain Pathol..

[89] Godbout J.P., Johnson R.W. (2009). Age and Neuroinflammation: A Lifetime of Psychoneuroimmune Consequences. Immunol. Allergy Clin. N. Am..

[90] Costa J., Martins S., Ferreira P.A., Cardoso A.M.S., Guedes J.R., Peça J., Cardoso A.L. (2021). The old guard: Age-related changes in microglia and their consequences. Mech. Ageing Dev..

[91] Savage J.C., Carrier M., Tremblay M. (2019). Morphology of Microglia Across Contexts of Health and Disease. Methods Mol. Biol..

[92] Shahidehpour R.K., Higdon R.E., Crawford N.G., Neltner J.H., Ighodaro E.T., Patel E., Price D., Nelson P.T., Bachstetter A.D. (2021). Dystrophic microglia are associated with neurodegenerative disease and not healthy aging in the human brain. Neurobiol. Aging.

[93] Jurgens H.A., Johnson R.W. (2012). Dysregulated neuronal-microglial cross-talk during aging, stress and inflammation. Exp. Neurol..

[94] VanGuilder H.D., Bixler G.V., Brucklacher R.M., Farley J.A., Yan H., Warrington J.P., Sonntag W.E., Freeman W.M. (2011). Concurrent hippocampal induction of MHC II pathway components and glial activation with advanced aging is not correlated with cognitive impairment. J. Neuroinflamm..

[95] Mela V., Mota B.C., Milner M., McGinley A., Mills K.H.G., Kelly Á., Lynch M.A. (2020). Exercise-induced re-programming of age-related metabolic changes in microglia is accompanied by a reduction in senescent cells. Brain Behav. Immun..

[96] Mouton P.R., Long J.M., Lei D.L., Howard V., Jucker M., Calhoun M.E., Ingram D.K. (2002). Age and gender effects on microglia and astrocyte numbers in brains of mice. Brain Res..

[97] Nichols N.R. (1999). Glial responses to steroids as markers of brain aging. J. Neurobiol..

