# Supplementary material for: Unraveling the Influence of Litter Size, Maternal Care, Exercise, and Aging on Neurobehavioral Plasticity and Dentate Gyrus Microglia Dynamics in Male Rats
Source: Brain Sci. 2024 May 15;14(5):497. doi: 10.3390/brainsci14050497 (PMC11119659; doi:10.3390/brainsci14050497)
Supplement: Supplementary file 1 [file brainsci-14-00497-s001.zip › Table S1.pdf]

Table S1. Microglial granular layer estimates for aged, exercised and sedentary rats raised in large and small litters. Experimental parameters, optical fractionator counting results and individual unilateral microglial numbers (N) and mean groups with the coefficient of error (CE).

| *Subjects* | *Section thickness (µm)* | *N* | *CE* | *tsf* | *No. of counting frames* | *$\Sigma Q^-$* | *Subjects* | *Section thickness (µm)* | N | *CE* | *tsf* | *No. of counting frames* | *$\Sigma Q^-$* |
|---|---|---|---|---|---|---|---|---|---|---|---|---|---|
| **Aged Sedentary from Large Litters** | | | | | | | **Aged Exercised from Large Litters** | | | | | | |
| ***SMG20 EX62*** | 31.5 ± 7.55 | 7354.49 | 0.070 | 0.258± 0.037 | 198 | 126 | ***PAE G13*** | 17.5 ± 0.14 | 5088.23 | 0.063 | 0.400 ± 0.003 | 220 | 151 |
| ***VIE G21 EX66*** | 23.5 ± 0.22 | 8337.7 | 0.059 | 0.298 ± 0.003 | 206 | 184 | ***SM G13*** | 40.2 ± 0.81 | 6841.19 | 0.083 | 0.175 ± 0.003 | 213 | 89 |
| ***VSDE G21 EX64*** | 32.7 ± 5.33 | 9083.46 | 0.0615 | 0.236 ± 0.034 | 199 | 146 | ***SM G32*** | 30.5 ± 3.11 | 6241.22 | 0.081 | 0.241 ± 0.025 | 184 | 108 |
| ***VSDE G29EX119*** | 26.7 ± 0.39 | 7679.83 | 0.068 | 0.263± 0.004 | 201 | 150 | ***VIE G32 A*** | 24.9 ± 1.14 | 5440.69 | 0.073 | 0.284 ± 0.012 | 203 | 114 |
| ***VSDEG29EX120*** | 32.2 ± 3.10 | 5493.52 | 0.085 | 0.224 ± 0.017 | 200 | 90 | ***VSE G32 A*** | 27.5 ± 1.44 | 4986.48 | 0.084 | 0.257 ± 0.013 | 210 | 94 |
| ***Mean*** | 29.3± 1.80 | 7589.8 | 0.069 | | | | ***Mean*** | 28.1 ± 3.7 | 5719.6 | 0.077 | | | |
| ***SD*** | | 1346.12784 | | | | | ***SD*** | | 797.4859 | | | | |
| ***CV$^2$=(SD/Mean)$^2$*** | | 0.031 | | | | | ***CV$^2$=(SD/Mean)$^2$*** | | 0.019 | | | | |
| ***CE$^2$*** | | 0.005 | | | | | ***CE$^2$*** | | 0.006 | | | | |
| ***CE$^2$/CV$^2$*** | | **0.1507** | | | | | ***CE$^2$/CV$^2$*** | | 0.3032 | | | | |
| ***CVB$^2$*** | | **0.027** | | | | | ***CVB$^2$*** | | 0.014 | | | | |
| ***CVB$^2$ (% of CV$^2$)*** | | 85 | | | | | ***CVB$^2$ (% of CV$^2$)*** | | 70 | | | | |
| **Aged Sedentary from Small Litters** | | | | | | | **Aged Exercised from Small Litters** | | | | | | |
| ***DOR EXP 122*** | 27.6 ± 4.06 | 4501.15 | 0.077 | 0.284± 0.049 | 155 | 88 | SMG23EX56 | 22.2±0.71 | 4345.33 | 0.075 | 0.317 ± 0.01 | 203 | 100 |
| ***SM G01B*** | 18.1 ± 0.06 | 4644.68 | 0.071 | 0.388 ± 0.001 | 171 | 134 | VIEG23EX58 | 21.4±1.21 | 4493.32 | 0.072 | 0.333 ± 0.019 | 195 | 110 |
| ***VME G04B*** | 19.2 ± 0.40 | 4642.5 | 0.071 | 0.366 ± 0.007 | 194 | 125 | VSDG01A | 18.7±0.62 | 4277.57 | 0.076 | 0.376 ± 0.011 | 196 | 117 |
| ***VSD G04B*** | 21.5 ± 0.69 | 4862.41 | 0.068 | 0.327 ± 0.011 | 173 | 118 | VSEG23EX59 | 24.4±1.04 | 4738.17 | 0.080 | 0.289 ± 0.012 | 196 | 99 |
| ***VSE G01*** | 20.2 ± 0.34 | 4112.38 | 0.074 | 0.348 ± 0.006 | 190 | 106 | VSEG25 | 22.1±0.76 | 4506.42 | 0.079 | 0.319 ± 0.011 | 223 | 105 |
| ***Mean*** | 21.3 ± 1.66 | 4552.62 | 0.072 | | | | Mean | 21.8±0.91 | **4472.16** | 0.076 | | | |
| ***S.D.*** | | 277.93 | | | | | S.D. | | 177.72 | | | | |
| ***CV$^2$=(D.P./Mean)$^2$*** | | 0.004 | | | | | CV$^2$=(D.P./Mean)$^2$ | | 0.002 | | | | |
| ***CE$^2$*** | | 0.005 | | | | | CE$^2$ | | 0.006 | | | | |
| ***CE$^2$/CV$^2$*** | | 1.3958 | | | | | CE$^2$/CV$^2$ | | 3.668 | | | | |
| ***CVB$^2$*** | | -0.001 | | | | | CVB$^2$ | | -0.004 | | | | |
| ***CVB$^2$ (% of CV$^2$)*** | | -40 | | | | | CVB$^2$ (% of CV$^2$) | | -267 | | | | |

[a]All evaluations were performed using a 100X objective lens (Nikon, NA 1.3, DF = 0.19µm). a(frame), area of the optical dissector counting frame = 60 x 60 µm$^2$; A(x,y step), x and y step sizes = 90 x 90; asf, area sampling fraction [a(frame)/A(x,y step)] = 0.44; tsf, thickness sampling fraction, calculated by the height of optical dissector = 7µm divided by section thickness, h/section thickness; ssf, section sampling fraction = 1/6; number of sections = 5; $\sum Q^-$, counted microglial markers.
