# Supplementary material for: Unraveling the Influence of Litter Size, Maternal Care, Exercise, and Aging on Neurobehavioral Plasticity and Dentate Gyrus Microglia Dynamics in Male Rats
Source: Brain Sci. 2024 May 15;14(5):497. doi: 10.3390/brainsci14050497 (PMC11119659; doi:10.3390/brainsci14050497)
Supplement: Supplementary file 1 [file brainsci-14-00497-s001.zip › Table S2.pdf]

Table S2. Microglial granular layer estimates for mature, exercised and sedentary rats raised in large and small litters. Experimental parameters, optical fractionator counting results and individual unilateral microglial numbers (N) and mean groups with the coefficient of error (CE).

Subjects	***Section thickness (µm)***	***N***	***CE***	***tsf***	***No. of counting frames***	***ΣQ^-***	***Subjects***	***Section thickness (µm)***	*N*	***CE***	***tsf***	***No. of counting frames***	***ΣQ^-***
**Mature Sedentary from Large Litters**							**Mature Exercised from Large Litters**						
SM G39 EXP 96	20.4 ± 1.07	6845.66	0.063	0.349 ± 0.02	204	174	***CAB G56 EXP 143***	17.9 ± 0.39	3683.76	0.075	0.392 ± 0.008	195	107
VIDE G38 EXP 86	19.2 ± 1.09	4337.82	0.073	0.372 ± 0.02	193	117	***DOR G56 EXP 142***	18.8 ± 0.59	4990.08	0.063	0.374 ± 0.011	185	138
VIE G39 EXP 94	21.8 ± 0.96	4777.27	0.074	0.325 ± 0.02	183	114	***PPE G56 EXP 144***	20.1 ± 0.72	3948.6	0.082	0.350 ± 0.013	188	102
VSD G38 EXP 89	19.7 ± 0.87	5219.27	0.064	0.359 ± 0.01	189	137	***VIDE G41 EXP 105***	24.1 ± 0.46	4425.97	0.078	0.291 ± 0.006	183	96
VSE+VID G39 EXP 92	19.9 ± 1.75	4554.58	0.072	0.362 ± 0.03	197	126	***VME G47 EXP 106***	21.3 ± 1.38	5058.2	0.070	0.335 ± 0.023	191	124
Mean	20.2 ± 0.44	**5146.92**	0.069				***Mean***		**4421.32**	0.074			
SD		1004.129					***SD***		611.7				
CV^2=(SD/Mean)^2		0.038					***CV^2=(SD/Mean)^2***		0.019				
CE^2		0.005					***CE^2***		0.005				
CE^2/CV^2		**0.1257**					***CE^2/CV^2***		0.2826				
CVB^2		0.033					***CVB^2***		0.014				
CVB^2 (% of CV^2)		87%					***CVB^2 (% of CV^2)***		72				
**Mature Sedentary from Small Litters**							**Mature Exercised from Small Litters**						
PAD G52 EXP 136	15.6 ± 0.35	3193.78	0.075834	0.450 ± 0.010	181	106	***DOR G51 EXP 126***	22.9 ± 0.98	3861.76	0.086	0.301 ± 0.014	214	87
PPE G52 EXP 135	13.9 ± 0.90	3322.62	0.076046	0.515 ± 0.034	200	123	***CAB G32 EXP 124***	23.2 ± 0.31	3417.89	0.095	0.303 ± 0.004	195	76
SM G32 EXP 148	15.5 ± 0.37	3090.66	0.079999	0.453 ± 0.011	186	103	***VID G37 EXP 70***	18.5 ± 1.15	3875.78	0.078	0.386 ± 0.027	185	108
SM G52 EXP 134	18.9 ± 0.82	4153.19	0.1	0.375 ± 0.015	206	113	***VMD EXP 52***	19.0 ± 0.89	3348.55	0.08	0.374 ± 0.018	193	93
VSDE G37 EXP 71	14.8 ± 0.68	3928.22	0.067753	0.479 ± 0.021	187	137	***VME G36 EXP 67***	19.3 ± 1.36	4112.68	0.074	0.369 ± 0.022	193	112
Mean	15.7 ± 0.84	**3537.694**	0.079926				***Mean***	20.6 ± 1.01	**3723.332**	0.082			
S.D.		473.21					***S.D.***		327.01				
CV^2=(D.P./Mean)^2		0.018					***CV^2=(D.P./Mean)^2***		0.002				
CE^2		0.006					***CE^2***		0.007				
CE^2/CV^2		0.3570					***CE^2/CV^2***		0.8812				
CVB^2		0.011					***CVB^2***		0.001				
CVB^2 (% of CV^2)		64.3%					***CVB^2 (% of CV^2)***		12				

[a]All evaluations were performed using a 100X objective lens (Nikon, NA 1.3, DF = 0.19µm). a(frame)‚ area of the optical dissector counting frame = 60 x 60 µm^2; A(x,y step), x and y step sizes = 90 x 90; asf, area sampling fraction [a(frame)/A(x,y step)] = 0.44; tsf, thickness sampling fraction, calculated by the height of optical dissector = 7µm divided by section thickness, h/section thickness; ssf, section sampling fraction = 1/6; number of sections = 5; $\sum Q^-$, counted microglial markers.
