# Supplementary material for: Unraveling the Influence of Litter Size, Maternal Care, Exercise, and Aging on Neurobehavioral Plasticity and Dentate Gyrus Microglia Dynamics in Male Rats
Source: Brain Sci. 2024 May 15;14(5):497. doi: 10.3390/brainsci14050497 (PMC11119659; doi:10.3390/brainsci14050497)
Supplement: Supplementary file 1 [file brainsci-14-00497-s001.zip › Table S3.pdf]

Table S3. Microglial molecular layer estimates for aged, exercised and sedentary subjects raised in large and small litters. Experimental parameters, optical fractionator counting results and individual unilateral microglial numbers (N) and mean groups with the coefficient of error (CE).

Subjects	*Section thickness (µm)*	*N*	*CE*	*tsf*	*No. of counting frames*	*ΣQ^-*	*Subjects*	*Section thickness (µm)*	*N*	*CE*	*tsf*	*No. of counting frames*	*ΣQ^-*
**Aged Sedentary from Large Litters**							**Aged Exercised from Large Litters**						
SMG20 EX62	31.8 ± 7.43	31315.52	0.047	0.253 ± 0.036	199	305	PAE G13	17.7 ± 0.38	26842.82	0.038	0.396 ± 0.0083	224	443
VIE G21 EX66	23.4 ± 0.24	35491.54	0.040	0.299 ± 0.003	213	439	SM G13	40.2 ± 0.65	28099	0.052	0.175 ± 0.0028	212	203
VSDE G21 EX64	33.1 ± 5.21	36923.36	0.044	0.233 ± 0.034	203	338	SM G32	30.5 ± 3.55	27355.25	0.050	0.244 ± 0.0281	210	266
VSDE G29EX119	26.7 ± 0.41	32348.03	0.045	0.262 ± 0.004	203	351	VIE G32 A	25.8 ± 1.64	22590.08	0.047	0.277 ± 0.0163	198	258
VSDEG29EX120	32.4 ± 1.80	34783.94	0.045	0.219 ± 0.011	216	311	VSE G32 A	28.1 ± 1.47	24445.39	0.049	0.252 ± 0.0132	211	255
Mean	29.5± 1.89	**34172.48**	0.044				***Mean***	25866.51	**25866.51**	0.047			
SD		2300.65108					***SD***		2286.27				
CV^2=(SD/Mean)^2		0.005					***CV^2=(SD/Mean)^2***		0.008				
CE^2		0.002					***CE^2***		0.002				
CE^2/CV^2		**0.4288**					***CE^2/CV^2***		0.2865				
CVB^2		**0.003**					***CVB^2***		0.006				
CVB^2 (% of CV^2)		57					***CVB^2 (% of CV^2)***		71				
**Aged Sedentary from Small Litters**							**Aged Exercised from Small Litters**						
DOR EXP 122	29.5 ± 3.89	29682.22	0.043	0.262 ± 0.047	197	304	SMG23EX56	22.7 ± 0.26	22578.74	0.045	0.309 ± 0.036	211	289
SM G01B	18.6 ± 0.31	24145.84	0.039	0.378 ± 0.006	198	380	VIEG23EX58	22.1 ± 1.50	21724.54	0.048	0.323 ± 0.021	210	293
VME G04B	18.9 ± 0.29	21524.84	0.044	0.370 ± 0.006	207	330	VSDG01A	18.4 ± 0.61	23877.26	0.040	0.382 ± 0.012	215	378
VSD G04B	21.6 ± 0.64	22572.45	0.046	0.325 ± 0.010	212	304	VSEG23EX59	24.8 ± 1.17	22193.94	0.051	0.285 ± 0.013	209	261
VSE G01	19.8 ± 0.58	21415.26	0.045	0.356 ± 0.010	211	316	VSEG25	22.4 ± 0.59	23503.34	0.043	0.314 ± 0.008	228	305
Mean	21.7 ± 2.02	**23868.122**	0.044				Mean		**22775.56**	0.045			
S.D.		3430.27445					S.D.		897.9239				
CV^2=(D.P./Mean)^2		0.021					CV^2=(D.P./Mean)^2		0.002				
CE^2		0.002					CE^2		0.002				
CE^2/CV^2		**0.0921**					CE^2/CV^2		**1.3170**				
CVB^2		**0.019**					CVB^2		**0.000**				
CVB^2 (% of CV^2)		91					CVB^2 (% of CV^2)		-32				

[a]All evaluations were performed using a 100X objective lens (Nikon, NA 1.3, DF = 0.19µm). a(frame), area of the optical dissector counting frame = 60 x 60 μm^2; A(x,y step), x and y step sizes = 90 x 90; asf, area sampling fraction [a(frame)/A(x,y step)] = 0.44; tsf, thickness sampling fraction, calculated by the height of optical dissector = 7µm divided by section thickness, h/section thickness; ssf, section sampling fraction = 1/6; number of sections = 5; $\sum Q^-$, counted microglial markers.
