# Supplementary material for: Unraveling the Influence of Litter Size, Maternal Care, Exercise, and Aging on Neurobehavioral Plasticity and Dentate Gyrus Microglia Dynamics in Male Rats
Source: Brain Sci. 2024 May 15;14(5):497. doi: 10.3390/brainsci14050497 (PMC11119659; doi:10.3390/brainsci14050497)
Supplement: Supplementary file 1 [file brainsci-14-00497-s001.zip › Table S4.pdf]

Table S4. Microglial molecular layer estimates for mature, exercised and sedentary rats raised in large and small litters. Experimental parameters, optical fractionator counting results and individual unilateral microglial numbers (N) and mean groups with the coefficient of error (CE).

Subjects	*Section thickness (µm)*	*N*	*CE*	*tsf*	*No. of counting frames*	*ΣQ^-*
**Mature Sedentary from Large Litters**						
SM G39 EXP 96	21.2 ± 1.55	30981	0.05	0.341 ± 0.031	241	426
VIDE G38 EXP 86	19.7 ± 1.09	21664.67	0.05	0.363 ± 0.019	208	322
VIE G39 EXP 94	22.5 ± 1.16	27878.07	0.04	0.316 ± 0.017	211	368
VSD G38 EXP 89	19.6 ± 0.69	34254.02	0.03	0.360 ± 0.013	228	509
VID G39 EXP 92	20.3 ± 1.23	26981.75	0.04	0.342 ± 0.020	205	395
Mean	20.7 ± 0.54	**28351.9**	0.04			
SD		4705.53				
CV^2=(SD/Mean)^2		0.028				
CE^2		0.002				
CE^2/CV^2		0.0703				
CVB^2		0.026				
CVB^2 (% of CV^2)		93%				
**MatureSedentary from Small Litters**						
PAD G52 EXP 136	16.0 ± 0.44	16082.51	0.045	0.441 ± 0.013	213	291
PPE G52 EXP 135	14.1 ± 0.92	16272.66	0.051	0.506 ± 0.033	226	327
SM G32 EXP 148	15.9 ± 0.19	15834.46	0.049	0.442 ± 0.005	201	292
SM G52 EXP 134	19.4 ± 0.98	24809.92	0.038	0.365 ± 0.018	268	373
VSDE G37 EXP 71	15.8 ± 0.92	18657.07	0.048	0.452 ± 0.026	216	340
Mean	16.2 ± 0.86	**18331.32**	0.046			
S.D.		3794.99				
CV^2=(D.P./Mean)^2		0.043				
CE^2		0.002				
CE^2/CV^2		0.0499				
CVB^2		0.041				
CVB^2 (% of CV^2)		95%				

Subjects	*Section thickness (µm)*	N	*CE*	*tsf*	*No. of counting frames*	*ΣQ^-*
**Mature Exercised from Large Litters**						
CAB G56 EXP 143	19.1 ± 0.58	25352.66	0.04	0.36946 ± 0.0109	247	388
DOR G56 EXP 142	19.6 ± 0.89	26975.39	0.04	0.36086 ± 0.0158	223	399
PPE G56 EXP 144	21.2 ± 0.94	28779.58	0.04	0.33401 ± 0.0159	215	399
VIDE G41 EXP 105	24.4 ± 0.42	26054.13	0.04	0.28773 ± 0.0049	204	310
VME G47 EXP 106	21.9 ± 1.66	29130.15	0.04	0.32774 ± 0.0258	225	389
Mean	21.2± 0.94	**27258.4**	0.04			
SD		1656.78				
CV^2=(SD/Mean)^2		0.004				
CE^2		0.002				
CE^2/CV^2		0.5060				
CVB^2		0.002				
CVB^2 (% of CV^2)		49%				
**Mature Exercised from Small Litters**						
DOR G51 EXP 126	23.4 ± 1.01	21531.2	0.05	0.304 ± 0.014	236	268
CAB G32 EXP 124	23.9 ± 0.28	20991.13	0.05	0.297 ± 0.004	234	255
VID G37 EXP 70	19.4 ± 1.43	21096.16	0.05	0.370 ± 0.031	224	320
VMD EXP 52	19.8 ± 0.77	17486.28	0.05	0.359 ± 0.014	224	254
VME G36 EXP 67	20.0 ± 1.49	23311.8	0.05	0.358 ± 0.023	217	344
Mean	21.3 ± 0.96	**20883.3**	0.05			
S.D.		2116.25				
CV^2=(D.P./Mean)^2		0.010				
CE^2		0.002				
CE^2/CV^2		0.2387				
CVB^2		0.008				
CVB^2 (% of CV^2)		76%				

[a]All evaluations were performed using a 100X objective lens (Nikon, NA 1.3, DF = 0.19µm). a(frame), area of the optical dissector counting frame = 60 x 60 μm^2; A(x,y step), x and y step sizes = 90 x 90; asf, area sampling fraction [a(frame)/A(x,y step)] = 0.44; tsf, thickness sampling fraction, calculated by the height of optical dissector = 7µm divided by section thickness, h/section thickness; ssf, section sampling fraction = 1/6; number of sections = 5; $\sum Q^-$, counted microglial markers.
