# Supplementary material for: Unraveling the Influence of Litter Size, Maternal Care, Exercise, and Aging on Neurobehavioral Plasticity and Dentate Gyrus Microglia Dynamics in Male Rats
Source: Brain Sci. 2024 May 15;14(5):497. doi: 10.3390/brainsci14050497 (PMC11119659; doi:10.3390/brainsci14050497)
Supplement: Supplementary file 1 [file brainsci-14-00497-s001.zip › Table S5.pdf]

Table S5. Microglial polymorphic layer estimates for aged, exercised and sedentary rats raised in large and small litters. Experimental parameters, optical fractionator counting results and individual unilateral microglial numbers (N) and mean groups with the coefficient of error (CE).

Subjects	Section thickness (µm)	N	CE	tsf	No. of counting frames	ΣQ⁻	Subjects	Section thickness (µm)	N	CE	tsf	No. of counting frames	ΣQ⁻
**Aged Sedentary from Large Litters**							**Aged Exercised from Large Litters**						
SMG20 EX62	32.4 ± 7.69	27768.73	0.045	0.249 ± 0.035	208	265	PAE G13	18.2 ± 0.29	21137.91	0.044	0.386 ± 0.006	216	338
VIE G21 EX66	24.4 ± 0.30	30496.62	0.041	0.288 ± 0.003	200	363	SM G13	41.1 ± 0.60	23926.4	0.053	0.170 ± 0.003	211	168
VSDE G21 EX64	33.9 ± 5.18	33614.91	0.042	0.227 ± 0.032	200	302	SM G32	33.5 ± 3.12	23285.19	0.063	0.222 ± 0.023	205	205
VSDE G29EX119	27.1 ± 0.28	33714.75	0.048	0.258 ± 0.002	214	362	VIE G32 A	26.2 ± 1.21	18720.87	0.052	0.269 ± 0.012	205	208
VSDEG29EX120	33.4 ± 2.57	26736.54	0.052	0.214 ± 0.014	215	234	VSE G32 A	29.3 ± 1.30	20622.89	0.051	0.241 ± 0.010	204	206
Mean	30.2± 1.89	30466.31	0.046				Mean	29.7 ± 3.80	21538.65	0.053			
SD		3227.03754					SD		2103.024				
$CV^2=(SD/Mean)^2$		0.011					$CV^2=(SD/Mean)^2$		0.010				
CE^2		0.002					CE^2		0.003				
CE^2/CV^2		**0.1861**					CE^2/CV^2		0.2918				
CVB^2		**0.009**					CVB^2		0.007				
CVB^2 (% of CV^2)		81%					CVB^2 (% of CV^2)		71				
**Aged Sedentary from Small Litters**							**Aged Exercised from Small Litters**						
DOR EXP 122	30.4 ± 3.91	18690.85	0.053	0.257 ± 0.049	161	187	SMG23EX56	22.2±0.71	19418.85	0.048	0.310 ± 0.007	206	254
SM G01B	19.3 ± 0.20	15951.26	0.055	0.364 ± 0.004	165	239	VIEG23EX58	21.4±1.21	19125.14	0.046	0.309 ± 0.012	223	248
VME G04B	19.4 ± 0.52	16177.82	0.050	0.362 ± 0.009	191	243	VSDG01A	18.7±0.62	18840.02	0.044	0.378 ± 0.005	214	294
VSD G04B	22.2 ± 0.72	17137.98	0.047	0.317 ± 0.011	212	227	VSEG23EX59	24.4±1.04	20141.05	0.046	0.275 ± 0.007	207	229
VSE G01	21.2 ± 0.33	21281.11	0.042	0.331 ± 0.005	219	290	VSEG25	22.1±0.76	17835.57	0.045	0.307 ± 0.015	213	226
Mean	22.5 ± 2.04	17847.8	0.049				Mean	22.5 ± 1.11	**19072.13**	0.046			
S.D.		2201.58423					S.D.		843.88				
$CV^2=(D.P./Mean)^2$		0.015					$CV^2=(D.P./Mean)^2$		0.002				
CE^2		0.002					CE^2		0.0021				
CE^2/CV^2		0.1593					CE^2/CV^2		1.0675				
CVB^2		0.013					CVB^2		-0.0001				
CVB^2 (% of CV^2)		84%					CVB^2 (% of CV^2)		-6.7453				

[a]All evaluations were performed using a 100X objective lens (Nikon, NA 1.3, DF = 0.19µm). a(frame), area of the optical dissector counting frame = 60 x 60 µm^2; A(x,y step), x and y step sizes = 120 x 120; asf, area sampling fraction [a(frame)/A(x,y step)] = 0.25; tsf, thickness sampling fraction, calculated by the height of optical dissector = 7µm divided by section thickness, h/section thickness; ssf, section sampling fraction = 1/6; number of sections = 5; ∑Q⁻, counted microglial markers.
