# Supplementary material for: Unraveling the Influence of Litter Size, Maternal Care, Exercise, and Aging on Neurobehavioral Plasticity and Dentate Gyrus Microglia Dynamics in Male Rats
Source: Brain Sci. 2024 May 15;14(5):497. doi: 10.3390/brainsci14050497 (PMC11119659; doi:10.3390/brainsci14050497)
Supplement: Supplementary file 1 [file brainsci-14-00497-s001.zip › Table S6.pdf]

Table S6. Microglial polymorphic layer estimates for mature, exercised and sedentary rats raised in large and small litters. Experimental parameters, optical fractionator counting results and individual unilateral microglial numbers (N) and mean groups with the coefficient of error (CE).

Subjects	*Section thickness (µm)*	*N*	*CE*	*tsf*	*No. of counting frames*	*ΣQ⁻*
**Mature Sedentary from Large Litters**						
SM G39 EXP 96	21.0 ± 0.95	22123.43	0.044	0.338 ± 0.017	207	305
VIDE G38 EXP 86	19.6 ± 1.06	16861.75	0.047	0.363 ± 0.018	198	253
VIE G39 EXP 94	22.2 ± 0.96	20119.39	0.050	0.318 ± 0.033	207	263
VSD G38 EXP 89	19.0 ± 0.57	21918.56	0.043	0.370 ± 0.011	209	336
VID G39 EXP 92	20.8 ± 1.79	18787.87	0.048	0.347 ± 0.028	203	274
Mean	20.5 ± 0.56	**19962.2**	0.046			
SD		2208.778				
CV^2=(SD/Mean)^2		0.012				
CE^2		0.002				
CE^2/CV^2		0.1739				
CVB^2		0.010				
CVB^2 (% of CV^2)		83				
**MatureSedentary from Small Litters**						
PAD G52 EXP 136	16.3 ± 0.30	11971.18	0.054	0.441 ± 0.013	202	214
PPE G52 EXP 135	14.5 ± 0.91	13099.32	0.047	0.506 ± 0.033	200	259
SM G32 EXP 148	15.3 ± 0.29	11548.52	0.050	0.442 ± 0.005	201	220
SM G52 EXP 134	18.9 ± 0.92	15869.08	0.046	0.365 ± 0.018	222	243
VSDE G37 EXP 71	15.6 ± 0.75	14771.88	0.047	0.452 ± 0.026	212	275
Mean	16.1 ± 0.75	**13452**	0.049			
S.D.		1838.804				
CV^2=(D.P./Mean)^2		0.019				
CE^2		0.002				
CE^2/CV^2		0.1268				
CVB^2		0.016				
CVB^2 (% of CV^2)		87				

Subjects	*Section thickness (µm)*	N	*CE*	*tsf*	*No. of counting frames*	*ΣQ⁻*
**Mature Exercised from Large Litters**						
CAB G56 EXP 143	19.0 ± 0.64	16844.02	0.048	0.371 ± 0.012	211	259
DOR G56 EXP 142	19.5 ± 0.56	21365.45	0.042	0.361 ± 0.010	211	320
PPE G56 EXP 144	20.4 ± 0.68	23465.51	0.042	0.345 ± 0.011	202	328
VIDE G41 EXP 105	24.5 ± 0.32	20521.48	0.047	0.286 ± 0.003	218	242
VME G47 EXP 106	21.5 ± 1.45	20123.41	0.048	0.332 ± 0.023	209	278
Mean	20463.97	**20463.97**	0.045			
SD		2400.273				
CV^2=(SD/Mean)^2		0.014				
CE^2		0.002				
CE^2/CV^2		0.1497				
CVB^2		0.012				
CVB^2 (% of CV^2)		85				
**Mature Exercised from Small Litters**						
DOR G51 EXP 126	24.0 ± 1.11	12888.41	0.065	0.296 ± 0.015	216	156
CAB G32 EXP 124	24.4 ± 0.35	14652.8	0.059	0.289 ± 0.004	210	174
VID G37 EXP 70	19.4 ± 0.97	16278.53	0.049	0.367 ± 0.019	212	248
VMD EXP 52	20.1 ± 0.91	14034.62	0.055	0.354 ± 0.017	208	204
VME G36 EXP 67	19.5 ± 1.26	15602.15	0.049	0.366 ± 0.022	210	235
Mean	21.5 ± 1.11	**14691.3**	0.055			
S.D.		1325.903				
CV^2=(D.P./Mean)^2		0.010				
CE^2		0.003				
CE^2/CV^2		0.3768				
CVB^2		0.005				
CVB^2 (% of CV^2)		62				

[a]All evaluations were performed using a 100X objective lens (Nikon, NA 1.3, DF = 0.19µm). a(frame), area of the optical dissector counting frame = 60 x 60 µm^2; A(x,y step), x and y step sizes = 120 x 120; asf, area sampling fraction [a(frame)/A(x,y step)] = 0.25; tsf, thickness sampling fraction, calculated by the height of optical dissector = 7µm divided by section thickness, h/section thickness; ssf, section sampling fraction = 1/6; number of sections = 5; ∑Q⁻, counted microglial markers.
